# Transcriptomic and hormonal dynamics in relation to adventitious rooting of two parental *Petunia* species highlight a coordinated activation of the jasmonate and auxin pathways and an important role of upper-shoot-derived auxin influx

**DOI:** 10.3389/fpls.2025.1707238

**Published:** 2026-02-06

**Authors:** Ivona Jurenic, Sindy Chamas, Nicole Nagler, Gerd Ulrich Balcke, Uwe Druege

**Affiliations:** 1 Erfurt Research Centre for Horticultural Crops (FGK), University of Applied Sciences Erfurt, Erfurt, Germany; 2 MetaCom Metabolomics Facility, Leibniz Institute of Plant Biochemistry, Halle (Saale), Germany

**Keywords:** cutting, wounding, adventitious root, phytohormone, auxin, jasmonate, ERF, petunia

## Abstract

**Introduction:**

Adventitious rooting of cuttings is a key developmental process for the vegetative propagation of many crops that involves phytohormone-controlled reprogramming and redifferentiation of specific cells in the stem base. The endogenous control of phytohormone action at the whole-plant level is not completely understood.

**Methods:**

Using the genome-sequenced *Petunia axillaris* and *Petunia inflata*, we monitored the transcriptome of phytohormone-related genes and phytohormone levels in different cutting sections through a phytohormone-targeted microarray, RT-qPCR, and LC-MS/MS, and analyzed the rooting response to manipulations of auxin levels and transport.

**Results:**

In the stem base of both species, genes controlling jasmonic acid (JA) biosynthesis, conjugation, and signaling, and encoding transcription factors of the ERF family were already upregulated at 0.5 hours post excision (hpe), followed by increased regulation of auxin-related genes. Accordingly, JA and its physiologically active isoleucine conjugate JA-Ile accumulated transiently at 0.5 hpe, before indole-3-acetic acid (IAA) peaked at 2 hpe. Genes controlling auxin biosynthesis were mostly downregulated, whereas three *IAA-leucine-resistant-like* genes were strongly upregulated between 0.5 and 2 hpe. *P. inflata’s* greater rooting capacity compared with *P. axillaris* was linked to higher stem-base IAA levels (0–72 hpe), resulting in a higher IAA/cytokinin ratio and stronger upregulation of auxin-signaling genes. *P. inflata* showed a steeper IAA gradient between the leaves and the stem base, which was positively and negatively correlated with leaf salicylic acid and cytokinin isopentenyladenine levels, respectively, and associated with exclusive upregulation of *PIN-like* genes in the leaves. *P. axillaris* showed a stronger improvement in rooting with low IAA doses than *P. inflata*. Blocking polar auxin transport in the upper shoot prevented rooting in both species.

**Discussion:**

The results reveal excision-triggered coordination of jasmonate and auxin pathways in the stem base, interacting with ERF transcription factors, and indicate an important role for upper shoot-derived auxin influx, potentially regulated by salicylic acid and cytokinins. Higher rooting capacity of *P. inflata* can be explained by the higher IAA level in the stem base. The results indicate important roles of *ERF113/114*, *ILR-like2 and 6*, *PIN6*, *PIN-like 1/3*, the *PINOID* gene *A4A49_10797*, *ARF11*, and several *LBD* genes in adventitious rooting of *Petunia*.

## Introduction

Adventitious root (AR) formation in cuttings is a key developmental process for the vegetative propagation of many horticultural and forestry crops that requires a reprogramming of particular responsive cells in the stem base near the wound and is evoked by two stimulating principles: wounding and isolation from the donor plant (reviewed in Druege et al., 2016 and Druege et al., 2019). It involves sequential phases. According to Da Costa et al. (2013) and Druege et al. (2019), three phases are distinguished. The first, the so-called induction phase, is characterized by an anatomical lag phase devoid of cellular changes, during which the initial cell reprogramming occurs. After the determination of AR founder cells, the initiation phase starts with qualitative changes in cell structures, followed by cell division and differentiation of the new cell clusters into dome-shaped root primordia. The final expression phase begins with the differentiation of primordia into the complete root body, with differentiated vascular bundles connected to the vascular cylinder of the stem, followed by the emergence of roots.

Research over several decades has shown that the rooting success of cuttings is dependent on primary metabolic processes but particularly on the action of plant hormones, while both factors are interrelated (reviewed in Da Costa et al., 2013; Druege et al., 2019; Lakehal and Bellini, 2019). Among the plant hormones, auxin plays an outstanding role. It acts as a positive regulator during the induction phase, whereas high auxin levels have an inhibitory role during subsequent differentiation and growth of ARs (Da Costa et al., 2013). Even though synthetic auxins have frequently been used to stimulate the rooting of cuttings, the major endogenous bottlenecks of auxin action at the whole-cutting level and the interactions with other plant hormones are not completely understood. Cytokinins act antagonistically against auxin during AR induction but, at low concentrations, have important functions during early cell reprogramming (reviewed in Da Costa et al., 2013). Some studies point to positive effects of ethylene during the early induction of ARs, possibly contributing to redifferentiation, while it also can interact with auxin in both directions (reviewed in Druege et al., 2019). Other studies suggest a positive regulator function of jasmonic acid (JA) during AR induction in cuttings (reviewed in Druege et al., 2019), which, however, stands in contrast to an inhibitory function of JA during etiolation-induced AR initiation in hypocotyls of intact *Arabidopsis* seedlings (Gutierrez et al., 2012).

Mostly, leafy shoot tip cuttings are used for vegetative propagation, which are not formed by the model plant *Arabidopsis*. Among horticultural plants, *Petunia hybrida* has an economically important role worldwide, and many cultivars are propagated by leafy shoot tip cuttings. *Petunia* has been established as a model for molecular investigations of diverse research questions (Gerats and Vandenbussche, 2005; Vandenbussche et al., 2016). We have previously used the model cultivar *P. hybrida* Mitchell and characterized the endogenous regulation of excision-induced AR formation. AR formation involves early accumulation of JA (Ahkami et al., 2009) and of indole-3-acetic acid (IAA) (Ahkami et al., 2013) in the stem base during the root induction phase. The accumulation of IAA and AR formation is dependent on a functioning polar auxin transport (PAT) in the cuttings, and PAT-controlled IAA accumulation is important not only for AR induction but also for the activation of invertases that control the establishment of the new sink in the stem base (Ahkami et al., 2013). Excision-induced AR formation of *P. hybrida* Mitchell involves differential expression of genes putatively controlling ethylene (ET), strigolactone (SL), and auxin homeostasis and signaling in the stem base, while rooting is dependent on ethylene biosynthesis and perception (Druege et al., 2014; Bombarely et al., 2016). The transcriptome data of these studies were limited as based on available ESTs and had not been linked to hormone data. In addition, the contribution of the upper cutting parts to excision-induced AR formation in the stem base of *Petunia* is still unknown.

Recently, the complete genomes of *Petunia axillaris* and *Petunia inflata*, which constitute important parental species of modern *P. hybrida* cultivars, have been sequenced (Bombarely et al., 2016). Preliminary studies indicated that both *P. axillaris* and *P. inflata* are easy to root, while *P. inflata* showed more intense rooting than *P. axillaris*.

In light of these findings and the still open questions, the present study aimed to address the following:

- Simultaneous analysis of the excision-induced dynamics of the phytohormone-related transcriptome and the phytohormone concentrations in the stem base and upper cutting sections of *P. axillaris* and *P. inflata.*
- Identification of important hormonal events and candidate genes that putatively control AR formation in both species and may contribute to the difference in rooting efficiency.

A phytohormone-targeted microarray, reverse transcription real-time quantitative polymerase chain reaction (RT-qPCR), and LC-MS/MS were combined with local pharmacological manipulations of auxin levels and transport to elucidate these relationships.

## Materials and methods

### Plant material, growth conditions, and analysis of rooting

**Figure 1:**
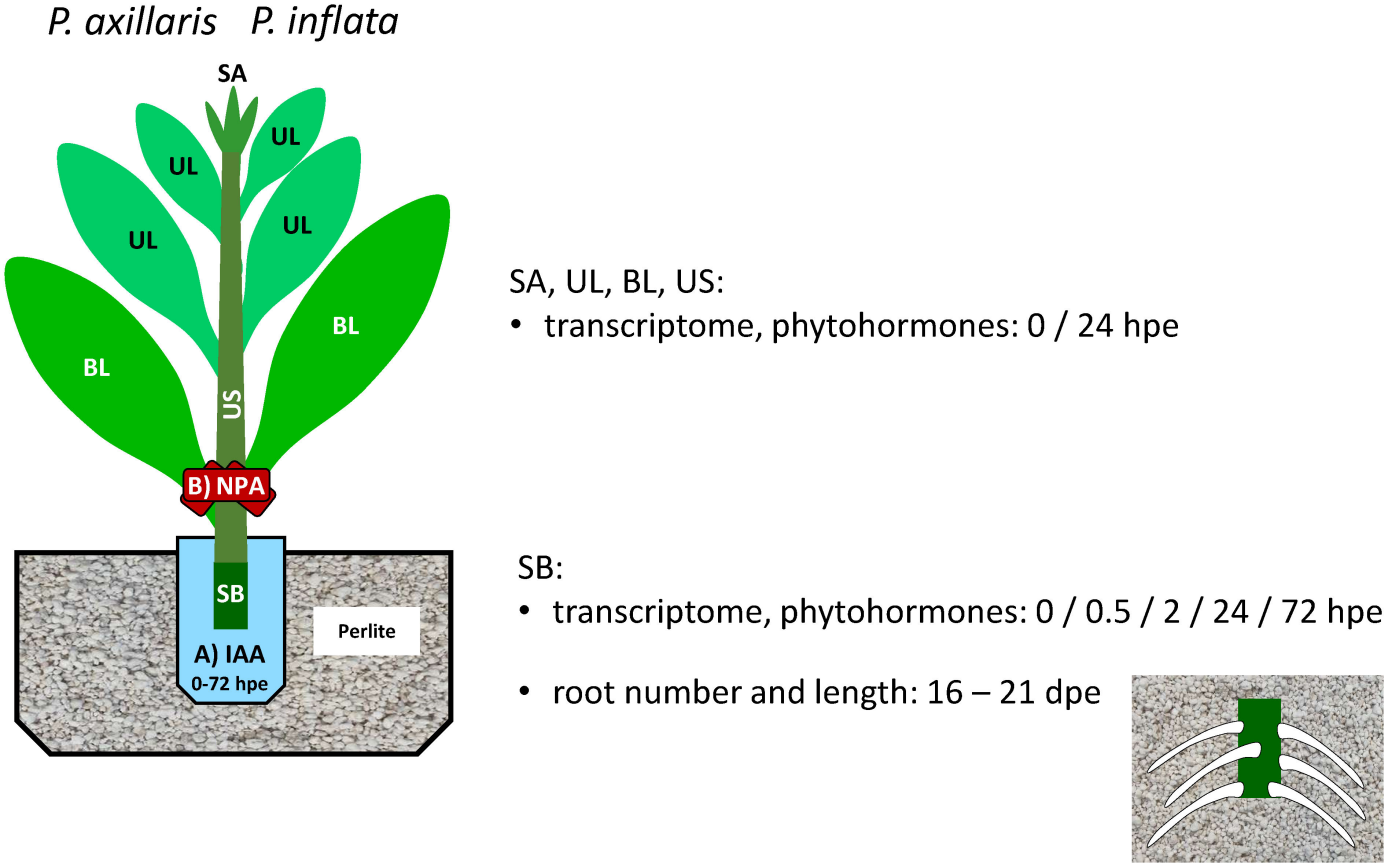
Experimental setup of transcriptome, phytohormone, and rooting analysis of cuttings of *P. axillaris* and *P. inflata*, and of IAA and NPA applications. SA, shoot apex including the smallest adjacent leaves that were maximally 1 cm in length; UL, upper leaves; BL, basal leaves; SB, stem base, 0.5 cm in length; US, upper stem, the stem section between SB and SA; hpe, hours post excision; dpe, days post excision.

Donor plants of *P. axillaris* N and *P. inflata* S6 were established from seeds and cultivated in the greenhouse as previously described for *P. hybrida* Mitchell (Klopotek et al., 2010). After the donor plants reached 3 months of age, shoot tip cuttings, as illustrated in **Figure 1**, were excised at regular intervals over a period of up to 6 months and used for the experiments, always leaving two leaves or nodes on the stock plant. Cuttings were planted in perlite Perligran A (Knauf Perlite GmbH, Dortmund, Germany) and cultivated in a growth chamber under the following conditions: temperature, 22°C/20°C (day/night); humidity outside the covered trays, 85%/60% (day/night); PPFD of 100 μmol m^−2^ s^−1^ at plant level during a 10-h photoperiod provided by white fluorescent tubes. During the rooting period, cuttings were manually watered and did not receive nutrients or phytohormones unless indicated otherwise (**Figure 1**). At specified days post excision (dpe) of the cuttings (**Figure 1**), ARs were counted and assigned to different root length classes (in 1-cm increments). The number of ARs formed per planted cutting, the mean length per AR, and the total root length per planted cutting were calculated as described by Druege et al. (2007) and Agulló-Antón et al. (2011).

### Sampling and grinding

At specified hours post excision (hpe) of the cuttings, according to **Figure 1**, their stem bases (SB, 0.5 cm in length), upper stems (US), longitudinal halves of the two basal leaves (BL) and the remaining upper leaves (UL), and shoot apices including the smallest adjacent leaves (SA) were shock-frozen in liquid nitrogen and stored at − 80°C. For each replicate (*n* = 3), pooled material from 10 or 16 cuttings was ground manually in liquid nitrogen using a mortar and pestle, and aliquots of 100 and 50 mg fresh mass were used for extraction and analysis of the transcriptome and the phytohormones, respectively.

### RNA extraction

Total RNA was extracted from the *Petunia* cutting samples following the protocol of the RNeasy Plant Mini Kit (Qiagen, Hilden, Germany). Up to 100 mg fresh mass was processed, according to the lysis capacity of the RLT buffer and RNeasy spin columns. Following the manufacturer’s protocol, 30 µL of total RNA was obtained. RNA integrity was checked on an agarose gel, and total RNA concentration and quality were determined using a NanoDrop™ 2000/2000c spectrophotometer (Thermo Fisher Scientific, Schwerte, Germany). Only high-quality RNA samples (OD260/280 = 1.8 − 2.2, OD260/230 ≥ 2) were used. The integrity of RNA used for microarray analysis was additionally verified using an Agilent 2100 Bioanalyzer (Agilent Technologies Deutschland GmbH, Waldbronn, Germany).

### Design and use of the phytohormone-targeted microarray

Based on previous findings on the hormonal regulation during AR formation in *P. hybrida* Mitchell, a phytohormone-targeted microarray was developed that covered those genes from *P. axillaris* and *P. inflata* that putatively control homeostasis, signal transduction, and downstream responses of/to auxin, JA, and SLs. To also cover the response of AR formation to ET, genes encoding ethylene response factors (ERFs) that may respond to ET and other phytohormones such as JA (Heyman et al., 2018) were included as the main gene family of the gene category “hormonal interaction” (short name “interaction”). To compile the gene list for the microarray, literature research was first carried out, and genes or gene families involved in the pathways mentioned above were selected. Subsequently, the *Petunia axillaris* v1.6.2 CDS and *Petunia inflata* v1.0.1 CDS databases (solgenomics.net) were searched for the respective annotated genes. Furthermore, the NCBI database was searched for homologous genes in other *Solanaceae* species, and the corresponding sequences (CDS or AAS) were blasted against the *Petunia* genomes. The results of the two BLAST searches were compared, and the gene list was adjusted accordingly. Later, based on the results of microarray, annotation of differentially expressed genes of the families *YUCCA family of flavin monooxygenase* (*YUCCA*), *IAA-leucine resistant* (*ILR*), *ILR-like* (*ILL*), *pin-formed* (*PIN*), *PIN-like*, *Auxin indole-3-acetic acid protein* (*Aux/IAA*), *auxin response factor* (*ARF*), *lateral organ boundaries domain* (*LBD*), *PROTEIN serine/threonine kinase pinoid* (*PINOID*), and *ERF* was updated using a new version of the *P. axillaris* genome 4.02 I, with the kind permission of Cris Kuhlemeier, University of Bern, Switzerland (later published as Pax403 under https://www.ncbi.nlm.nih.gov/datasets/genome/GCA_026929995.1/).

Based on the genome sequences of 1,265 genes of interest (605 from *P. axillaris* and 660 from *P. inflata*), an Array-to-Go Custom microarray was designed by OakLabs GmbH (Henningsdorf, Germany). For this purpose, five to 10 specific isothermal probes, each 45–60 bp in length, were designed for each gene of interest. An additional 1,000 probes from a previously used microarray (Yang et al., 2019) were used for normalization. The probes were spotted on a microarray. Extracted RNA samples were labeled with a Cy3 fluorescent dye and hybridized to the microarray, which was manufactured in collaboration with Agilent (Santa Clara, CA, USA). The best-performing oligonucleotide probe(s) were selected for each gene based on the hybridization data and used for the subsequent data analysis. Quantile-normalized expression values of the genes of interest were provided and then analyzed as described below.

### cDNA synthesis and reverse transcription real-time quantitative PCR analysis

Transcription of selected genes was additionally analyzed by RT-qPCR analysis. First-strand complementary DNA (cDNA) was synthesized from RNA using the PrimeScript RT Master Mix reverse transcriptase (Takara Bio Europe SAS, Saint-Germain-en-Laye, France) according to the manufacturer’s protocol. Four microliters of diluted RNA (125 ng µL^−1^) were added to 1 µL of 5× PrimeScript RT Master Mix. Primers, which are described in **Supplementary Table S1**, were selected using Primer3web version 4.1.0 software and obtained salt-free at 0.01 µM from Eurofins (Jena, Germany). RT-qPCR was performed in plates (Brand GmbH + Co KG, Wertheim) using iQ SYBR Green Supermix (Bio-Rad Laboratories GmbH, Feldkirchen, Germany) in a 10-µL reaction volume with three technical replicates per biological sample. The reaction contained 2.5 µL of primer mix, with 1/100 (Fw + Rv) diluted primers, 5 µL of SYBR Green, and 2.5 µL of diluted cDNA (1:20). Melting curve analysis confirmed the specificity and quality of the PCR products. RT-qPCR was conducted on the CFX96 Real-Time System (Bio-Rad) with an initial denaturation for 3 min at 95°C, followed by 40 cycles of 10 s at 95°C and 40 s at 60°C. After preliminary tests according to Mallona et al. (2010), *ribosomal protein S13* (*Ph-RPS13*) and *elongation factor 1-alpha* (*EF1α*) were used as reference genes. Cq values were calculated using the Bio-Rad CFX Maestro software. Relative transcript levels were determined by the 2^−ΔΔCT^ method (Livak and Schmittgen, 2001) with geometric averaging of the reference genes (Vandesompele et al., 2002).

### Plant hormone analyses

#### Hormone extraction

Fifty milligrams (fresh weight) of homogenate were extracted using three rounds with 80% methanol acidified to pH 2.4 with hydrochloric acid. For round 1, 400 µL; for round 2, 200 µL; and for round 3, 100 µL of solvent were used. Cell extraction was performed in 2 mL cryotubes with reinforced walls (710768, Biozym Scientific GmbH, Hessisch Oldendorf, Germany). To enhance cell rupture, one steel bead of 3 mm diameter, three steel beads of 1 mm diameter, and 200 mg glass beads with a diameter of 0.75–1 mm (Carl Roth GmbH, Karlsruhe Germany) were added to each tube, and bead milling was performed for 3 × 1 min in a homogenizer (FastPrep-24, MP Biomedicals, Eschwege, Germany). The combined extracts were pooled, centrifuged, and stored on ice until measurement on the same day.

#### Online SPE-UPLC-MS/MS phytohormone analysis

Hormones were analyzed by online solid-phase extraction coupled ultra-high-performance liquid chromatography–tandem mass spectrometry (SPE-UPLC–MS/MS) using a CTC Combi-PAL autosampler with a 1,000-µL injection loop, a 96-well rack of a CHROspe divinylbenzene polymer SPE phase (10 mm × 2 mm; particle size: 25–35 µm; fill weight, 10 mg), a SPH1299 UPLC gradient pump, an EPH30 UPLC dilution pump, a Mistral column oven (all Axel Semrau GmbH, Sprockhövel, Germany), and a mass spectrometer (QTrap 6500, ABSciex, Darmstadt, Germany). For each sample, 600 µL of plant extract was injected into the online SPE at a rate of 200 µL min^−1^, where phytohormones were trapped by simultaneous addition of excess water (3,800 µL min^−1^) before SPE. Sequential transfer of trapped hormones from the SPE cartridge to the UPLC column was accomplished by 120 µL of 20% acetonitrile and simultaneous water dilution prior to UPLC. For chromatographic separations by UPLC, a Nucleoshell RP Biphenyl column (100 mm × 2 mm × 2.7 µm, Macherey & Nagel, Düren, Germany) was used. The gradient was as follows: 0–2 min, 5% B; 2–13 min, linear gradient to 95% B; 13–15 min, 95% B; and 15–18 min, 5% B. The column temperature was 40°C, and the flow rate was 400 µL min^−1^. Solvent A was 0.3 mM ammonium formate, acidified with formic acid to pH 3.0, and solvent B was acetonitrile. The autosampler temperature was maintained at 4°C. Mass-spectrometric detection of the phytohormones on a QTrap 6500 (ABSciex) was accomplished by electrospray ionization and multiple reaction monitoring (MRM) using the parameters in **Supplementary Table S2**. For this, the ion source was heated to 450°C. Curtain gas was set to 35 psi, ion source GS1 to 60 psi, and GS2 to 70 psi. The electrospray ionization voltage was 5,500 V in positive mode and − 4,500 V in negative mode. Fully automated sample processing was controlled by Chronos (Axel Semrau GmbH) via contact closure to trigger MS/MS runs controlled by Analyst 1.7.1., based on protocols described by Balcke et al. (2012) and Ai et al. (2023).

### Pharmacological treatments

The rooting response of the two *Petunia* species to increasing external supply of IAA to the stem base during the root induction phase was analyzed under the same climate chamber conditions described above. For this purpose, cuttings, as described before, were immediately after excision placed in 25 mL vials (**Figure 1A**) containing one-half MS medium (Murashige and Skoog, 1962) supplemented with vitamins according to Gamborg et al. (1968) and adjusted to a pH of 5.8. The medium was supplemented with different concentrations of IAA (0, 5, 10, 50, and 150 mg L^−1^). The vials were sealed with paraffin to prevent water evaporation, with a small cut large enough for the cutting to pass through so that the stem base was immersed in the medium (**Figure 1A**). The cuttings were kept in the vials until 72 hpe and then replanted in hormone-free perlite and cultivated until rooting was assessed.

To test the dependency of AR formation in the stem base on PAT-derived auxin influx from the upper shoot of the cutting, including all leaves, immediately after excision, rings of lanolin paste (Carl Roth GmbH) containing 0, 10, or 30 mg L^−1^ 1-*N*-naphthylphthalamic acid (NPA; Duchefa, Haarlem, The Netherlands), were carefully applied around the petioles of the two basal leaves and the adjacent upper shoot, as illustrated in **Figure 1B**.

### Data analysis

Mean values and standard error (SE) at the experimental level, or standard deviation (SD) over several replicative experiments, were calculated for the rooting data, the expression values of specific genes analyzed by microarray and RT-qPCR, and the phytohormone levels. The microarray data were analyzed using the CLC Genomics Workbench (Qiagen, Hilden, Germany). The normalized transcript levels determined at 0.5, 2, 24, and 72 hpe (*x*) were related to the respective levels at 0 hpe (*y*), the time of excision, to calculate fold changes (FC). These fold changes were log-transformed to log2 FC (= log2 [*x*/*y*]). If the log2 FC values were > 2 or < −2 and the *p*-values of expression values were < 0.05 (*t*-test), the fold changes were considered significant. Numbers of up- and downregulated genes (DEGs) of different categories were counted. Ratios of DEGs were calculated based on the total number of respective genes covered by the microarray. Heat maps were developed to illustrate the intensity of regulation for specific genes. Root number and length, RT-qPCR and microarray data from selected genes, and phytohormone levels were further analyzed using Statistica Version 3, TIBCO Software Inc. (Palo Alto, CA, USA). Depending on the number of factor levels, the significance of differences was analyzed by *t*-test or by ANOVA followed by Tukey test (*p* < 0.05). Applied statistical tests and the number of replications (*n*) are provided with the data. Pearson correlation coefficients and slopes of linear regressions were determined to analyze interrelationships between specific data using the “trend” function of Microsoft Excel (Munich, Germany).

## Results

### Rooting capacity of *P. axillaris* and *P. inflata*

**Figure 2:**
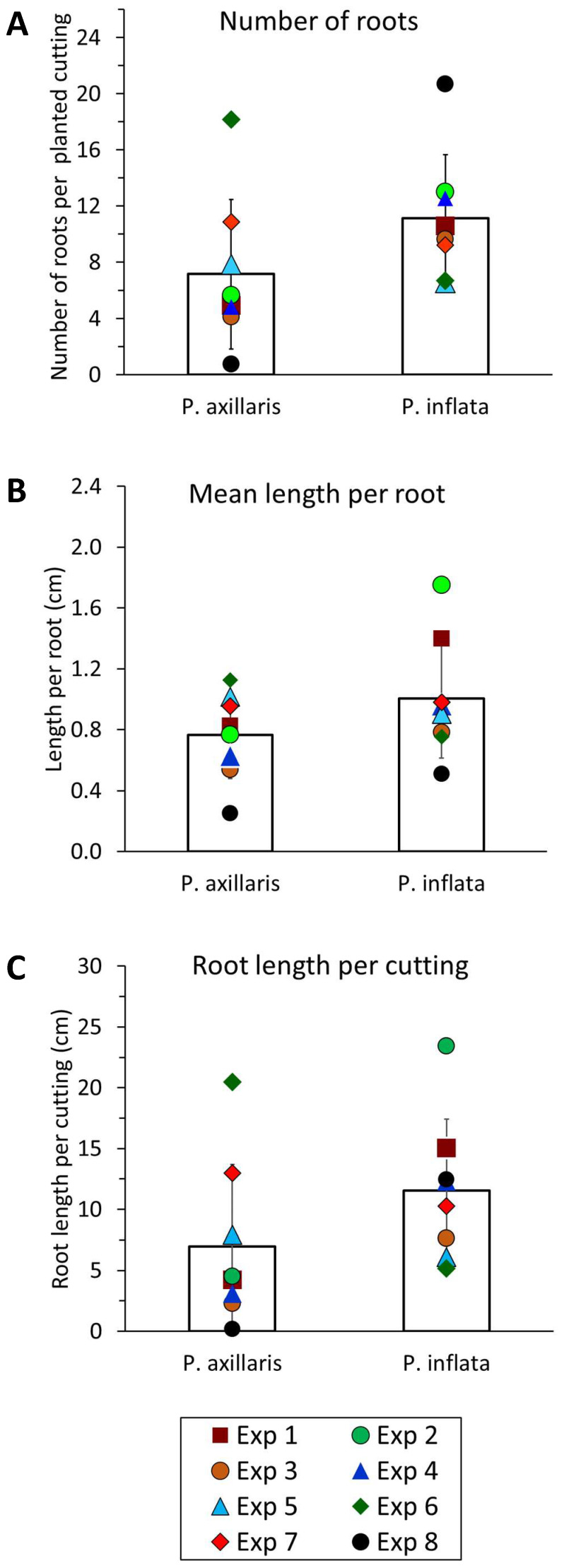
Rooting capacity of *P. axillaris* and *P. inflata*. The number of roots per planted cutting **(A)**, the mean length per root **(B)**, and the total root length per planted cutting **(C)** were determined in eight independent experiments (Exp). Roots were analyzed on day 16 (Exp 1–2, 4–6), day 17 (Exp 3, 8), and day 18 (Exp 7). Columns and whiskers represent the mean values ± standard deviation calculated over all experiments. Individual dots show the mean values per experiment. The experiments involved three (Exp 4–7) or four (Exp 1–3, 8) replications, each consisting of eight (Exp 3), 10 (Exp 4–8), or 12 (Exp 1, 2) cuttings.

**Figure 2** summarizes the results of eight independent experiments analyzing the rooting capacity of *P. axillaris* and *P. inflata*. The early detection of ARs on cuttings of both species after a period of 16 to 18 days post excision demonstrates that both *P. axillaris* and *P. inflata* are easy to root. However, higher mean values of root number (**Figure 2A**), mean root length (**Figure 2B**), and total root length (**Figure 2C**) were observed for *P. inflata* across the eight experiments when compared with *P. axillaris*. *P. inflata* produced at least six roots and a total root length of at least 5 cm, whereas the minimum number and total length of roots for *P. axillaris* were 1 and 0.5 cm, respectively. These results indicate a higher rooting capacity for *P. inflata* than for *P. axillaris.*

### Time course of expression of hormone-related genes in the stem base analyzed by a targeted microarray

To characterize the hormonal response of the two species to cutting excision, we first monitored the transcription of auxin-, JA-, and SL-related genes, as well as genes putatively controlling the interaction of these hormones, in the stem base of the cuttings using a phytohormone-targeted microarray. The intensive rooting of both species observed after 16 to 18 days (**Figure 2**) reflected a temporal progression of AR formation similar to that of histologically characterized *P. hybrida* Mitchell, which also showed intensive rooting after 16 days under the same conditions (Klopotek et al., 2010). Thus, a similar timing of the rooting phases in *P. axillaris* and *P. inflata* can be expected. According to the phase characterization of *P. hybrida* Mitchell (Klopotek et al., 2010), the transcriptome of the stem bases of *P. axillaris* and *P. inflata* was analyzed during the early induction phase (0.5 and 2 hpe), the central root induction phase (24 hpe), and the expected start of root initiation (72 hpe), and was compared with the respective transcriptome at 0 hpe, the time of excision. The lists, numbers, and ratios of up- and downregulated genes per species and time point for different gene categories and families are compiled in **Supplementary Table S3**.

**Figure 3:**
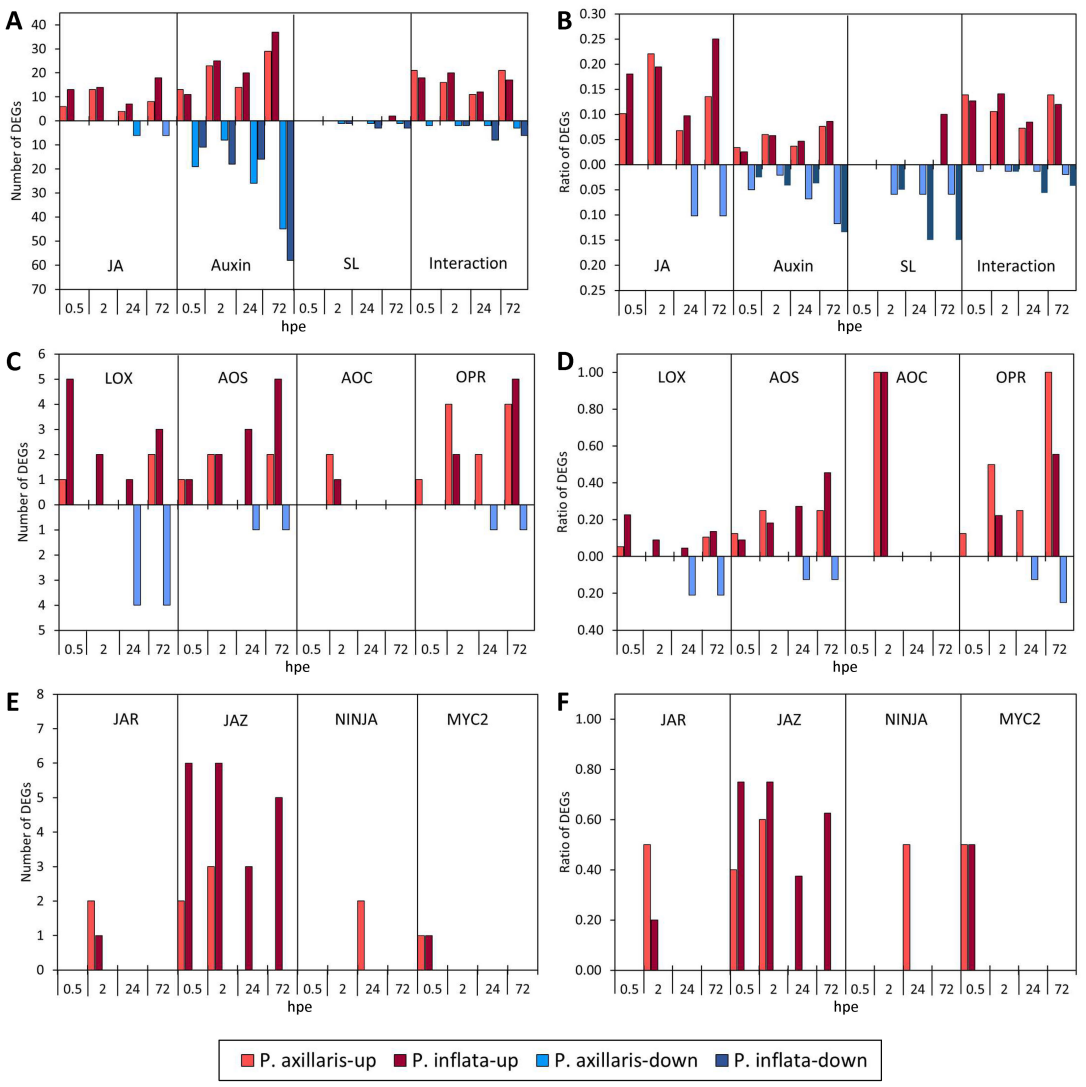
Number and ratio of up- and downregulated genes (DEGs) of different hormonal categories **(A, B)** and of JA-related genes **(C**–**F)** in the stem base of *P. axillaris* and *P. inflata* in response to cutting excision over time. Microarray data. Genes that showed a significantly different expression at the specified time point compared to the initial expression at 0 hours post excision (hpe) were counted. Criteria: *t*-test (*p* < 0.05) and log2 FC > 2 or < −2. Sample size (*n*) = 3, each sample consisting of material from 10 or 16 cuttings for 24 and 72 hpe or 0, 0.5, and 2 hpe, respectively.

**Figure 4:**
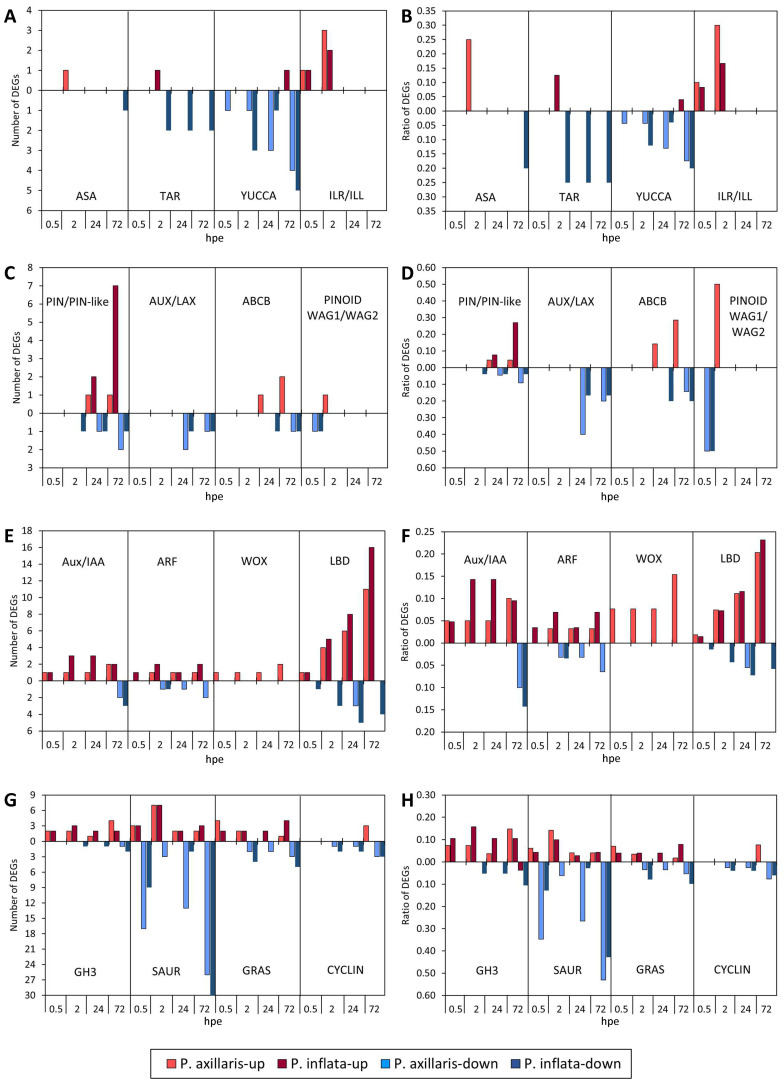
Number and ratio of up- and downregulated auxin-related genes (DEGs) of different categories **(A-H)** in the stem base of *P. axillaris* and *P. inflata* in response to cutting excision over time. Microarray data. Genes that showed a significantly different expression at the specified time point compared to the initial expression at 0 hours post excision (hpe) were counted. Criteria: *t*-test (*p* < 0.05) and log2 FC > 2 or < −2. Sample size (*n*) = 3, each sample consisting of material from 10 or 16 cuttings for 24 and 72 hpe or 0, 0.5, and 2 hpe, respectively.

The absolute and relative numbers of up- and downregulated genes of different hormonal categories in the stem base of the two species are illustrated in **Figures 3**, **4**. In both species, genes of the JA and hormonal interaction categories showed upregulation as early as 0.5 hpe and reached numbers at 2 hpe that were at or near the maximum level reached over the analyzed period. This was followed by a slight decline in the number of upregulated genes until 24 hpe, before the absolute and relative numbers increased again until 72 hpe (**Figures 3A, B**). *P. inflata* reached higher numbers and ratios of upregulated JA-related genes than *P. axillaris* at 0.5, 24, and 72 hpe. Only a few genes of the JA- and interaction category were downregulated. SL-related genes showed almost exclusive downregulation in both species. This applied to genes of the *carotenoid cleavage dioxygenase* (*CCD*) family and the *carotenoid isomerase* gene *D27* (**Supplementary Table S3**), which control SL biosynthesis (Al-Babili and Bouwmeester, 2015). The highest numbers of DEGs were found in the auxin category. In both species, the numbers of up- and downregulated auxin-related genes increased over time, reaching the highest values at 72 hpe. At 24 and 72 hpe, more auxin-related genes were upregulated in *P. inflata* than in *P. axillari*s. At 0.5 and 24 hpe, more auxin-related genes were downregulated in *P. axillaris* than in *P. inflata*, whereas the opposite pattern was found at 2 and 72 hpe. Considering the overall dynamics of gene transcription of the four hormonal categories, genes of the JA and interaction categories appeared as early responders, showing approximately the maximum number of upregulated genes already before the most comprehensive response of auxin-related genes was recorded. *Petunia inflata* displayed more frequent upregulation of JA- and auxin-related genes than *P. axillaris*.

Among the genes putatively controlling JA biosynthesis, genes of the *lipoxygenase* (*LOX*), the *allene oxide synthase* (*AOS*), the *allene oxide cyclase* (*AOC*), and the *12-oxophytodienoic acid* (OPDA) *reductase* (*OPR*) families were upregulated in both species at 0.5 and 2 hpe, and in *P. inflata*, also at the later time points (**Figures 3C, D**). In *P. inflata*, five and two *LOX* genes were upregulated at 0.5 and 2 hpe, respectively. By contrast, *P. axillaris* showed only one *LOX* gene upregulated at 0.5 hpe, but exhibited an exclusive downregulation of four *LOX* genes, one *AOS* gene, and one *OPR* gene at 24 and 72 hpe. At 2 hpe, both species showed upregulation of all *AOC* genes, two in *P. axillaris* and one in *P. inflata*. In *P. axillaris*, two genes that constituted 50% of the analyzed *JA-amino acid synthetase* (*JAR*) family and control the conversion of JA into the biologically active conjugate jasmonoyl-isoleucine (JA-Ile), were upregulated at 2 hpe, whereas in *P. inflata*, only one *JAR* gene showed such a response (**Figures 3E, F**). Corresponding to the higher number of upregulated *LOX* and *AOS* genes in *P. inflata*, this species also showed a higher number of upregulated *jasmonate zim domain* (*JAZ)* genes at all time points (**Figures 3E, F**). JAZ proteins function as repressors of JA signaling; however, their genes also belong to the early JA-responsive genes (Wasternack and Hause, 2013). One gene of the basic helix–loop–helix transcription factor myelocytomatosis 2 (MYC2), which acts as a positive regulator of the JA signaling and also belongs to the early JA-responsive genes, was upregulated at 0.5 hpe in each species, before two *novel interactor of JAZ* (*NINJA*) genes were upregulated at 24 hpe only in *P. axillaris*.

**Figure 5:**
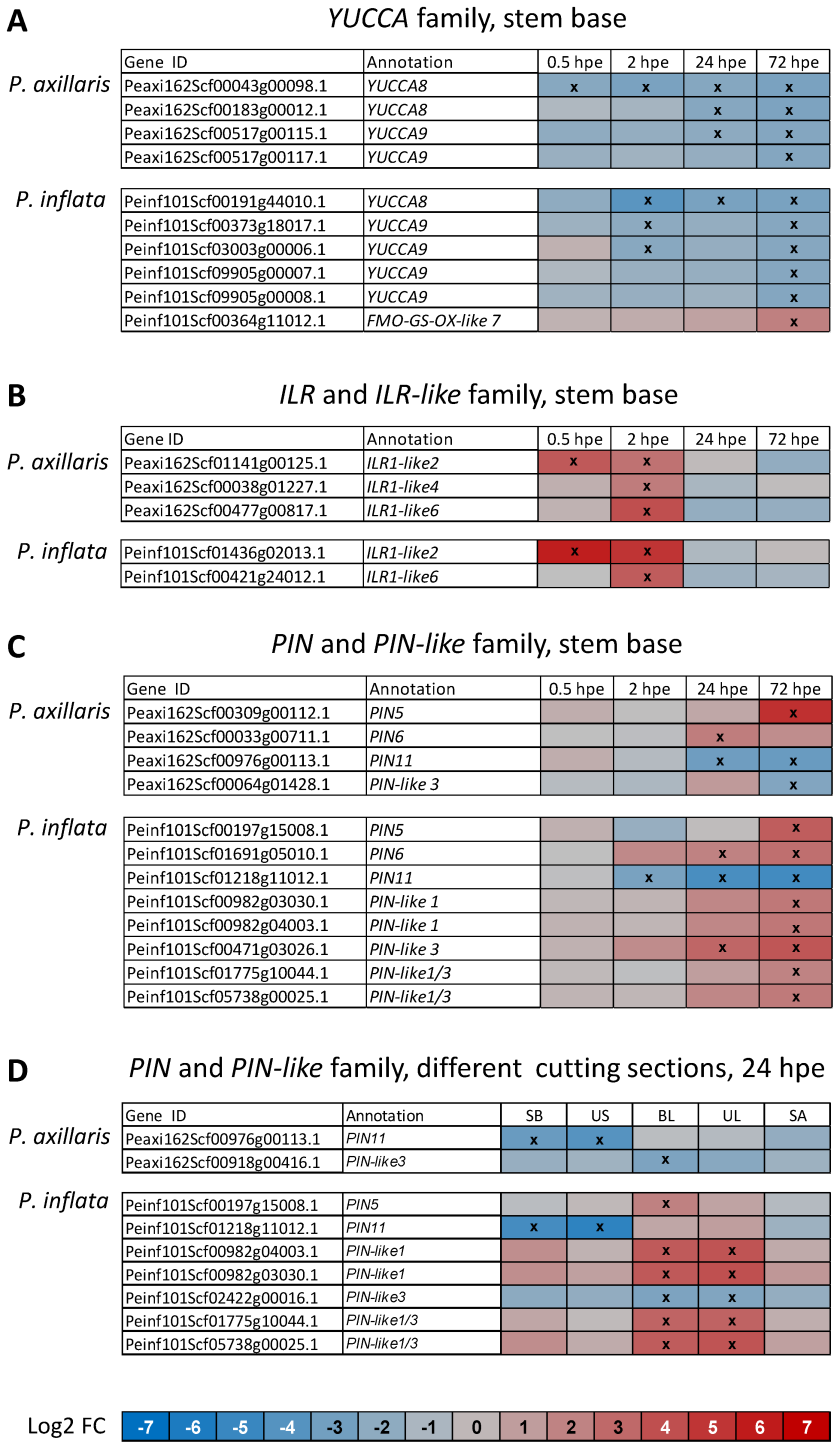
Heat maps showing the differential expression of genes of the *YUCCA* family **(A)**, the *ILL*-and *ILR-like* family **(B)**, and the *PIN* and *PIN-like* family **(C, D)** in the stem base **(A**–**C)** and in different cutting sections **(D)** of *P. axillaris* and *P. inflata* in response to cutting excision over time **(A**–**C)** or at 24 hours post excision (hpe) **(D)**. Microarray data. Crosses in the boxes indicate a significantly different expression at the specified time point compared to the initial expression at 0 hpe. Criteria: *t*-test (*p* < 0.05) and log2 FC > 2 or < −2. Sample size (*n*) = 3, each sample consisting of material from 10 or 16 cuttings for 24 and 72 hpe or 0, 0.5, and 2 hpe, respectively. UL, upper leaves; BL, basal leaves; SB, stem base, 0.5 cm in length; US, upper stem; SA, shoot apex.

Most genes that putatively control auxin biosynthesis were downregulated in both species after excision of the cuttings (**Figure 4A**). This applies to one *anthranilate synthase* (*ASA*) gene downregulated in *P. inflata* at 72 hpe, two *tryptophan amino transferase* (*TAR*) genes downregulated in *P. inflata* at 2, 24, and 72 hpe, and up to four and five *YUCCA* genes, downregulated in *P. axillaris* and *P. inflata*, respectively (**Figures 4A**, **5A**). The only exceptions from this response were the upregulation of one *ASA* gene in *P. axillaris* at 0.5 hpe and the upregulation of one *TAR* and one *YUCCA* gene (*FMO-GC-OX-like 7*) in *P. inflata* at 2 and 72 hpe, respectively (**Figures 4A**, **5A**). However, while auxin biosynthesis was mainly downregulated at the transcriptome level, excision of cuttings stimulated a fast and strong upregulation of *ILR*/*ILL* genes that encode IAA-amino acid amidohydrolases, in both species (**Figure 4A**). This applied to one *ILR1-like2* and one *ILR1-like6* gene in both species, and additionally to one *ILR1-like4* gene in *P. axillaris* (**Figure 5B**).

Excision of the cuttings modified the transcription of genes that control auxin transport in the stem base (**Figures 4C, D**, **5C**). Upregulation of *PIN* and *PIN-like* genes started at 24 hpe and involved higher gene numbers in *P. inflata* than in *P. axillaris*. The number of upregulated *PIN* and *PIN-like* genes in *P. inflata* even strongly increased between 24 and 72 hpe, whereas in *P. axillaris*, only one gene was upregulated at both 24 and 72 hpe. The upregulation involved *PIN5* and *PIN6* in both species, while five *PIN-like* genes were additionally upregulated in *P. inflata* (**Figure 5C**). In both species, upregulation of *PIN6* started at 24 hpe before *PIN5* was upregulated at the start of root initiation (72 hpe). *PIN11* was downregulated from 24 hpe onward in *P. axillaris* and from 2 hpe onward in *P. inflata*. *Auxin-resistant* (*AUX*)/*like AUX* (*LAX*) genes, which encode proteins controlling auxin influx, were exclusively downregulated in both species from 24 hpe onward (**Figures 4C, D**). At this time, more *AUX/LAX* genes were downregulated in *P. axillaris* than in *P. inflata*. Genes encoding the B family of membrane-bound ATP-binding cassette (ABC) transporters (ABCB) were preferentially upregulated in *P. axillaris* starting at 24 hpe, while *P. inflata* showed exclusive downregulation at 24 and 72 hpe. At 0.5 hpe, both species showed downregulation of one gene of the *PINOID/WAG1/WAG2* family, which may function in intracellular trafficking of PIN proteins.

**Figure 6:**
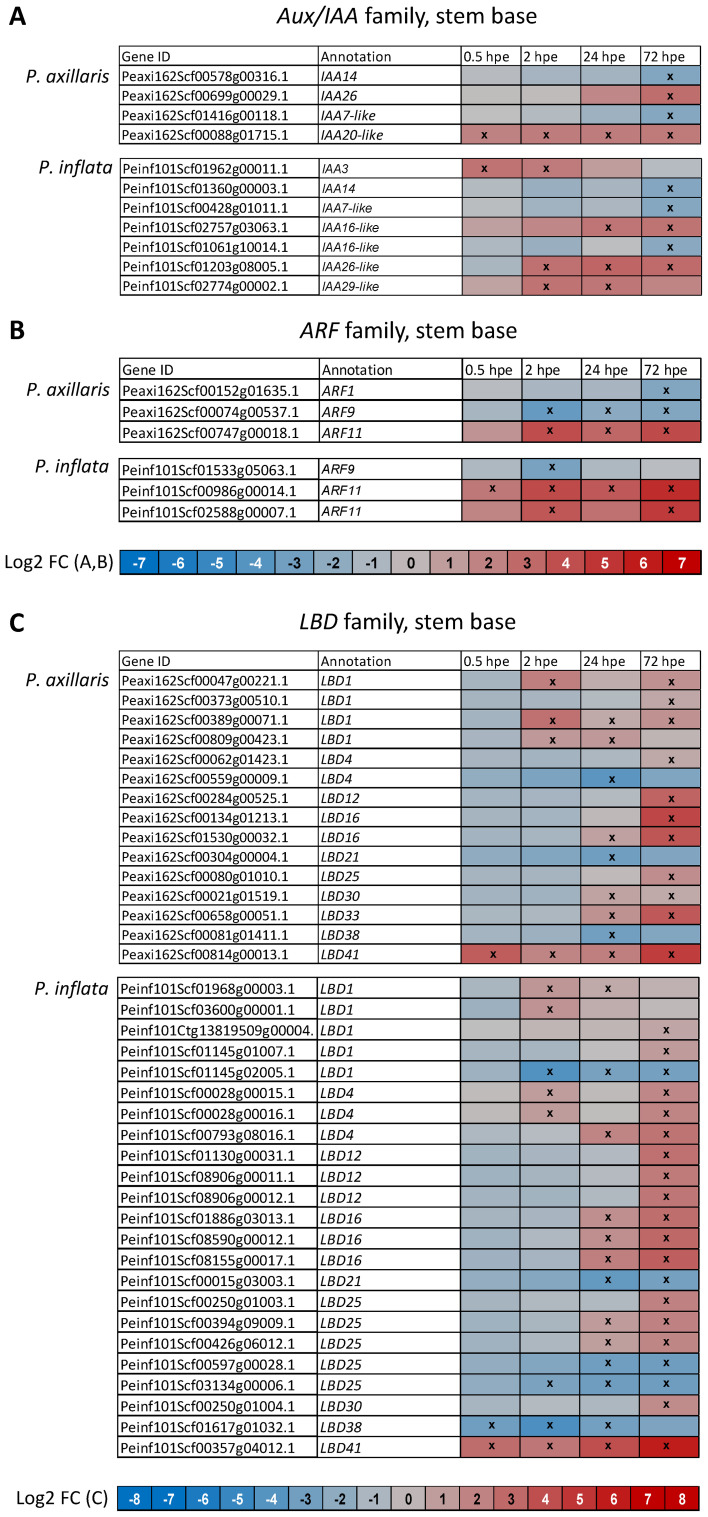
Heat maps showing the differential expression of genes of the *Aux/IAA* family **(A)**, the *ARF* family **(B)**, and the *LBD* family **(C)** in the stem base of *P. axillaris* and *P. inflata* in response to cutting excision over time. Microarray data. Crosses in the boxes indicate a significantly different expression at the specified time point compared to the initial expression at 0 hours post excision (hpe). Criteria: *t*-test (*p* < 0.05) and log2 FC > 2 or < −2. Sample size (*n*) = 3, each sample consisting of material from 10 or 16 cuttings for 24 and 72 hpe or 0, 0.5, and 2 hpe, respectively.

During the induction phase (0.5 until 24 hpe), expression of *Aux/IAA* genes that encode auxin repressor proteins was exclusively enhanced in both species (**Figures 4E, F**). At 2 and 24 hpe, the number and ratio of upregulated *Aux/IAA* genes were higher in *P. inflata* than in *P. axillaris*. At the start of root initiation (72 hpe), in addition to two upregulated *Aux/IAA* genes, two and three other *Aux/IAA* genes were downregulated in *P. axillaris* and *P. inflata*, respectively (**Figures 4E**, **6A**). Specific *ARF* genes were upregulated in both species after excision of cuttings, while in *P. inflata*, this gene family responded earlier and at 2 and 72 hpe involved a higher number and ratio of genes than in *P. axillaris* (**Figures 4E, F**). Downregulation of other *ARFs* was observed at 2, 24, and 72 hpe in *P. axillaris* but was restricted to 2 hpe in *P. inflata* (**Figures 4E, F**). One and two genes that showed homology to *ARF11* were upregulated from 2 hpe onward in *P. axillaris* and from 0.5 hpe onward in *P. inflata*, respectively, whereas one homologue of *ARF9* was downregulated in each species (**Figure 6B**).

While upregulation of two *WUSCHEL-related homeobox* (*WOX*) genes was found only in *P. axillaris*, both species revealed a comprehensive upregulation of genes of the *lateral organ boundaries domain* (*LBD*) family. The number of upregulated genes increased over time between 0.5 and 72 hpe and was higher in *P. inflata* than in *P. axillaris* (**Figures 4E**, **6C**). In parallel to the upregulation, fewer *LBD* genes were downregulated. In *P. inflata*, the number of downregulated genes increased between 0.5 and 72 hpe, while in *P. axillaris*, the downregulation was restricted to 24 hpe. In both species, genes showing high homology to *LBD41* were upregulated at each of the four time points, whereas genes showing homology to *LBD12* were upregulated exclusively at early root initiation (72 hpe). Genes of the *Gretchen Hagen 3* (*GH3*) family that may control conjugation of acidic hormones, particularly of IAA to amino acids, were preferentially upregulated in both species over the period from 0.5 to 72 hpe. At 2 and 24 hpe, the number and ratio of upregulated *GH3* genes were higher in *P. inflata* than in *P. axillaris* (**Figures 4G, H**). A large number and ratio of genes of the s*mall auxin up RNA* (*SAUR*) family were differentially expressed in the stem base after excision of the cuttings (**Figures 4G, H**). At 2 and 24 hpe, *P. axillaris* showed more downregulated *SAUR* genes than *P. inflata*. Genes of the *GAI*, *RGA*, and *SCR-like* (GRAS) family that may control auxin-induced cell reprogramming and differentiation at different levels were exclusively upregulated at 0.5 hpe, then reaching a higher gene number and ratio for *P. axillaris* (**Figures 4G, H**). At the following time points, both up- and downregulation were observed. At central root induction (24 hpe) and early root initiation (72 hpe), *P. inflata* showed a higher number and ratio of upregulated *GRAS* genes than *P. axillaris.* Two *Cyclin* genes that are thought to control cell division were upregulated at the time of root initiation (72 hpe) only in *P. axillaris*, whereas downregulation of other genes of this gene family was observed in both species between 2 and 72 hpe.

**Figure 7:**
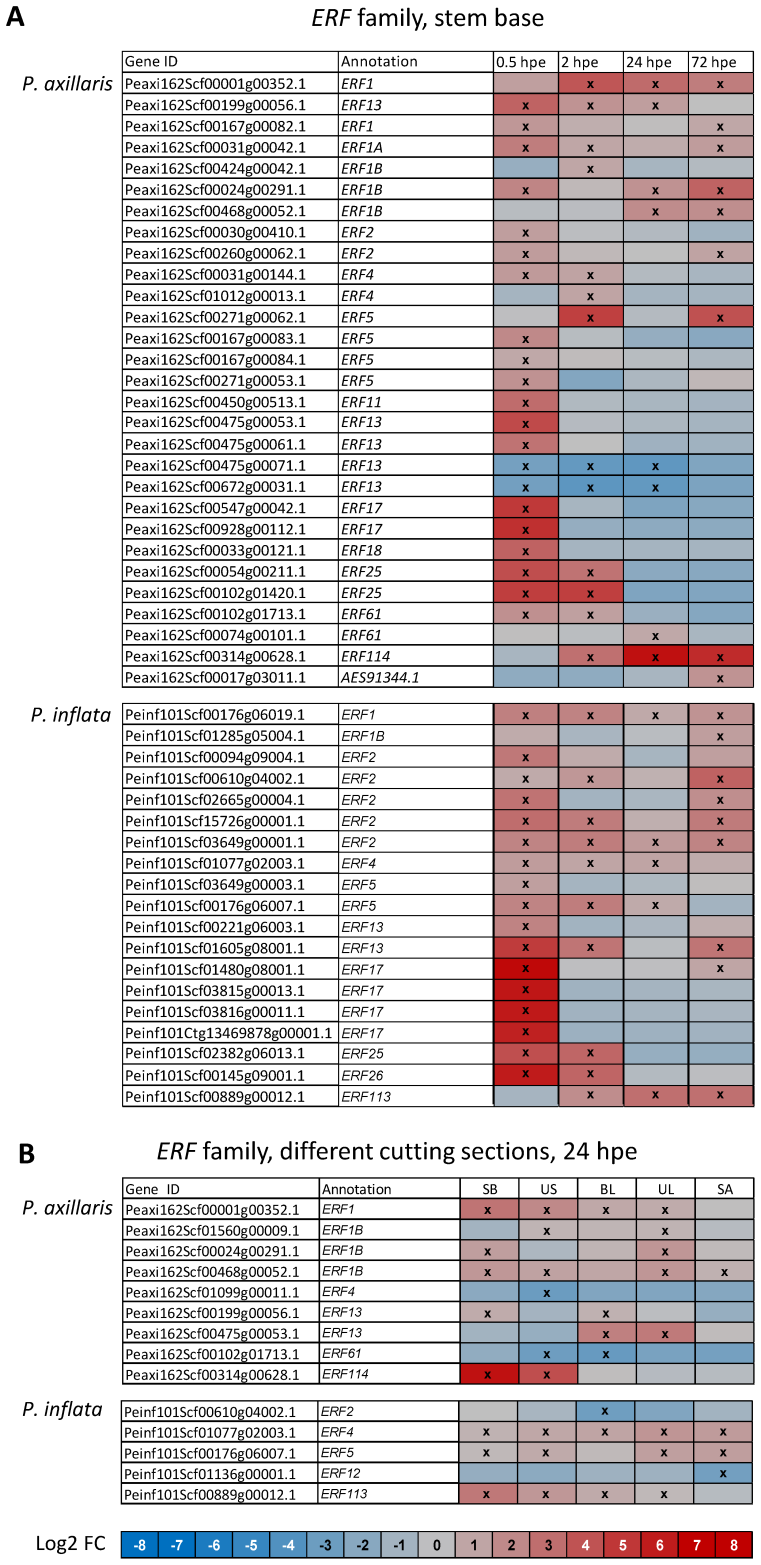
Heat maps showing the differential expression of genes of the *ERF* family in the stem base over time **(A)** and in different cutting sections at 24 hours post excision (hpe) **(B)** of *P. axillaris* and *P. inflata*. Microarray data. Crosses in the boxes indicate a significantly different expression at the specified time point compared to the initial expression at 0 hpe. Criteria: *t*-test (*p* < 0.05) and log2 FC > 2 or < −2. Sample size (*n*) = 3, each sample consisting of material from 10 or 16 cuttings for 24 and 72 hpe or 0, 0.5, and 2 hpe, respectively. UL, upper leaves; BL, basal leaves; SB, stem base, 0.5 cm in length; US, upper stem; SA, shoot apex.

Genes of the *ERF* family accounted for the largest proportion of regulated genes in the hormonal interaction category (**Figure 3A**). As illustrated in **Figure 7A**, 18 and 17 *ERF* genes were upregulated in the stem base of *P. axillaris* and *P. inflata* already at 0.5 hpe. Among these, genes with homology to *ERF17* were exclusively upregulated at this time point and reached higher magnitudes of upregulation in *P. inflata* than in *P. axillaris* (**Figure 7A**). During the following time points, upregulation of *ERF* genes became less pronounced. Among the upregulated genes, homologs of *ERF114* and *ERF113* that in *Arabidopsis* respond to JA and have already been related to etiolation-induced adventitious rooting (Lakehal et al., 2020) showed upregulation between 2 and 72 hpe in *P. axillaris* and *P. inflata*, respectively.

### Systemic effects of cutting excision on the expression of hormone-related genes as analyzed by a targeted microarray

**Figure 8:**
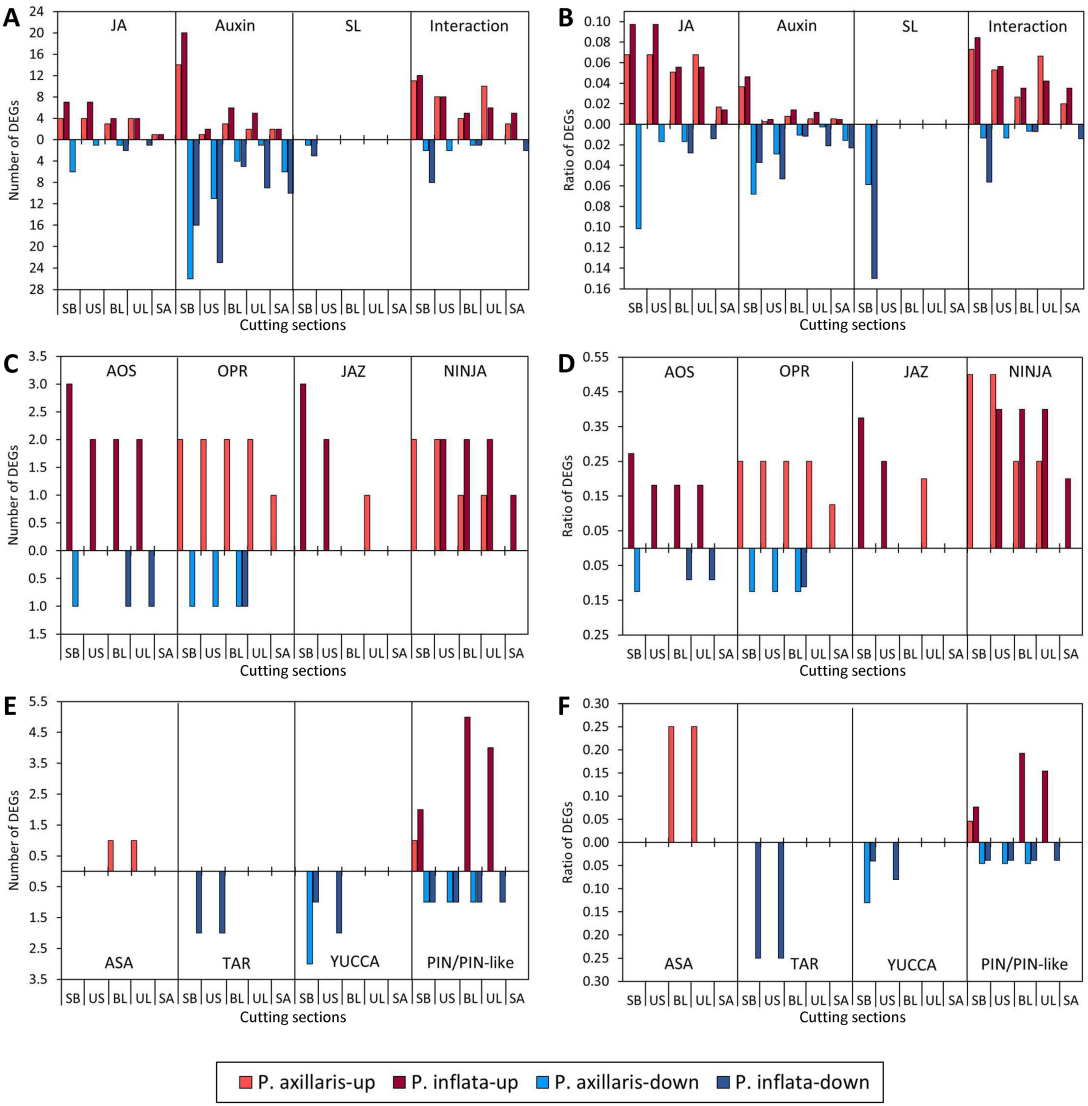
Number and ratio of up- and downregulated genes of different hormonal categories **(A-F)** in the stem base (SB), the upper stem (US), the two basal leaves (BL), the upper leaves (UL), and the shoot apex (SA) of *P. axillaris* and *P. inflata* at 24 hours post excision (hpe). Microarray data. Genes that showed a significantly different expression at the specified time point compared to the initial expression at 0 hpe were counted. Criteria: *t*-test (p < 0.05) and log2 FC > 2 or < −2. Sample size (*n*) = 3, each sample consisting of material from 10 or 16 cuttings for 24 hpe or 0 hpe, respectively.

At 24 hpe, the time point of central induction, expression of genes was also analyzed in upper cutting parts and related to the initial status at 0 hpe, when cuttings were excised (**Supplementary Table S4**, **Figure 8**). To illustrate the magnitude of the systemic effect in relation to the local effect, the data of the stem base are also included in **Figure 8**. The response of JA-, auxin-, and hormonal interaction-related genes to the excision of cuttings reached the shoot apex as the uppermost cutting part (**Figures 8A, B**). In the upper stem, the number of upregulated JA-related genes was even as high as in the stem base, with seven versus five upregulated genes for *P. inflata* compared to *P. axillaris.* A substantial upregulation of JA-related genes also occurred in upper and lower leaves, with three to four upregulated genes in each species. Almost no downregulation of JA-related genes was observed in the upper cutting parts. As was the case for the stem base, also in upper cutting sections, many DEGs were related to the auxin category (**Figure 8A**). Numbers and ratios of both up- and downregulated auxin-related DEGs were mostly higher in *P. inflata* than in *P. axillaris* (**Figures 8A, B**). In the latter, the pattern of upregulated genes revealed a strong decline from the stem base to a similarly low level for the upper stem, the upper and lower leaves, and the shoot apex. In *P. inflata*, a similar apical-directed decline of upregulated auxin-related genes was found, but in leaves, more than twice as many auxin-related genes were upregulated compared to *P. axillaris*. Similar to the upregulated genes, an apical-directed decline was also found for the downregulated auxin-related genes, which, however, was less strong. Regulation of SL-related genes was restricted to the stem base. The expression pattern of genes of the category hormonal interaction revealed only a weak gradient along the shoot axis, so that up to eight and 10 genes were upregulated in the upper cutting parts of *P. axillaris* and *P. inflata*, respectively. Almost no downregulation was observed for this category in the upper cutting parts.

Expression of genes controlling JA biosynthesis and signaling in the upper cutting parts responded to the excision of cuttings, but at different levels of the pathways for the two species (**Figures 8C, D**). In *P. inflata*, genes of the *AOS* family were mostly upregulated and also downregulated in all upper cutting sections except the shoot apex at frequencies similar to the stem base. *P. axillaris* showed no transcriptional response of *AOS* genes in the upper cutting parts to the excision, but upregulation and less downregulation of *OPR* genes in all upper cutting parts. Upregulation of *JAZ* was restricted to the upper stem in *P. inflata* and to the upper leaves of *P. axillaris*. Upregulation of *NINJA* genes was found in all upper tissues in *P. inflata* and in the upper stem and both leaf types in *P. axillaris*. Genes controlling auxin biosynthesis were mostly downregulated in the upper cutting parts in response to the cutting excision, except for one *ASA* gene that was upregulated in the leaves of *P. axillaris* (**Figures 8E, F**). By contrast, expression of auxin transporter genes revealed a species-dependent upregulation in cutting leaves. Five and four *PIN/PIN*-like genes were exclusively upregulated in basal and upper leaves of *P. inflata*, respectively, while one other gene of the same category was downregulated in all upper tissues of the same species and in the upper stem and the leaves of *P. axillaris*. It can be seen from **Figure 5D** that two genes with homology to *PIN-like1* and three other genes with homology to *PIN5*, *PIN-like1*, or *PIN-like3* were strongly upregulated only in the leaves of *P. inflata*. In each species, two other genes with homology to *PIN11* and to *PIN-like3* were downregulated in the upper stem and the leaves, respectively. *ERF* genes responded in the upper cutting parts to the excision of cuttings. Seven *ERF* genes in *P. axillaris* and three in *P. inflata* were upregulated in the upper cutting parts (**Figure 7B**). These included *ERF114* in *P. axillaris* and *ERF113* in *P. inflata* (**Figure 7B**). In each species, two other *ERF* genes showed downregulation in the upper cutting parts.

### Validation of the targeted microarray by RT-qPCR

Considering our focus on auxin, for validation of the microarray, we selected seventeen genes that control auxin biosynthesis, transport, signaling, and downstream response, analyzed the transcript levels in selected samples from *P. axillaris* or *P. inflata* by RT-qPCR, and compared the results with microarray data. High correspondence and significant correlation between both methods revealed the high quality of the microarray analysis (**Supplementary Figure S1**). Lower fold changes for *ASA2*, *IAA20-like*, and *SAUR68-like* determined by RT-qPCR, when compared to the microarray, even indicate that the microarray was more sensitive than RT-qPCR. This conclusion is supported by the expression levels determined by both methods (**Supplementary Figure S2**). These three genes, particularly *SAUR68-like*, revealed relatively low signal levels when analyzed by RT-qPCR.

### Plant hormone levels as affected by time after excision and cutting section

Corresponding to the analysis of transcription of genes putatively controlling plant homeostasis, signaling, and response, phytohormone concentrations were analyzed in the stem base of both species over time after excision and also in the upper cutting sections at 0 and 24 hpe, the time point of central root induction. In addition to the most important physiologically active auxin, IAA, JA, as well as its precursor OPDA, its physiological conjugate JA-Ile, and diverse cytokinins, which have important antagonistic functions against auxin, were analyzed. Furthermore, salicylic acid (SAL) was included because SAL has recently been found as a new hormonal player affecting AR formation in explants from *Arabidopsis* and cucumber while interacting with auxin (Dong et al., 2020; Tran et al., 2023). Strigolactones were not analyzed because SLs are extremely difficult to quantify in shoot tissues.

**Figure 9:**
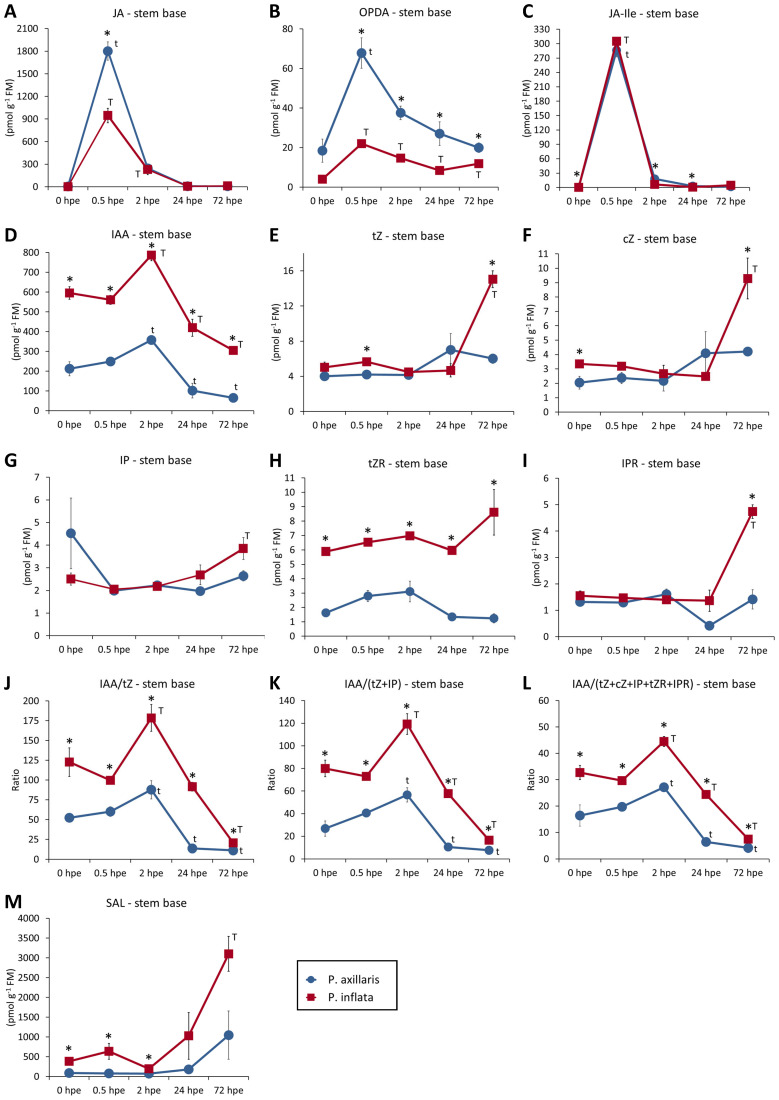
Concentrations of phytohormones in cutting parts of *P. axillaris* and *P. inflata*. Temporal course of JA **(A)**, OPDA **(B)**, JA-Ile **(C)**, IAA **(D)**, tZ **(E)**, cZ **(F)**, IP **(G)**, tZR **(H)**, IPR **(I)**, the ratio of IAA/tZ **(J)**, the ratio of IAA/(tZ + IP) **(K)**, the ratio of IAA/(tZ + cZ + IP + tZR + IPR) **(L)**, and the level of SAL **(M)** in the stem base. Asterisks indicate significant differences between the two species at the specified time points (*t*-test, *p* < 0.05). *t* and *T* indicate values that are significantly different from the values at 0 hours post excision (hpe) for *P. axillaris* and *P. inflata*, respectively (ANOVA, Tukey test, *p* < 0.05). Sample size (*n*) = 3, each sample consisting of material from 10 or 16 cuttings for 24 and 72 hpe or 0, 0.5, and 2 hpe, respectively. For abbreviations, see the text.

**Figure 10:**
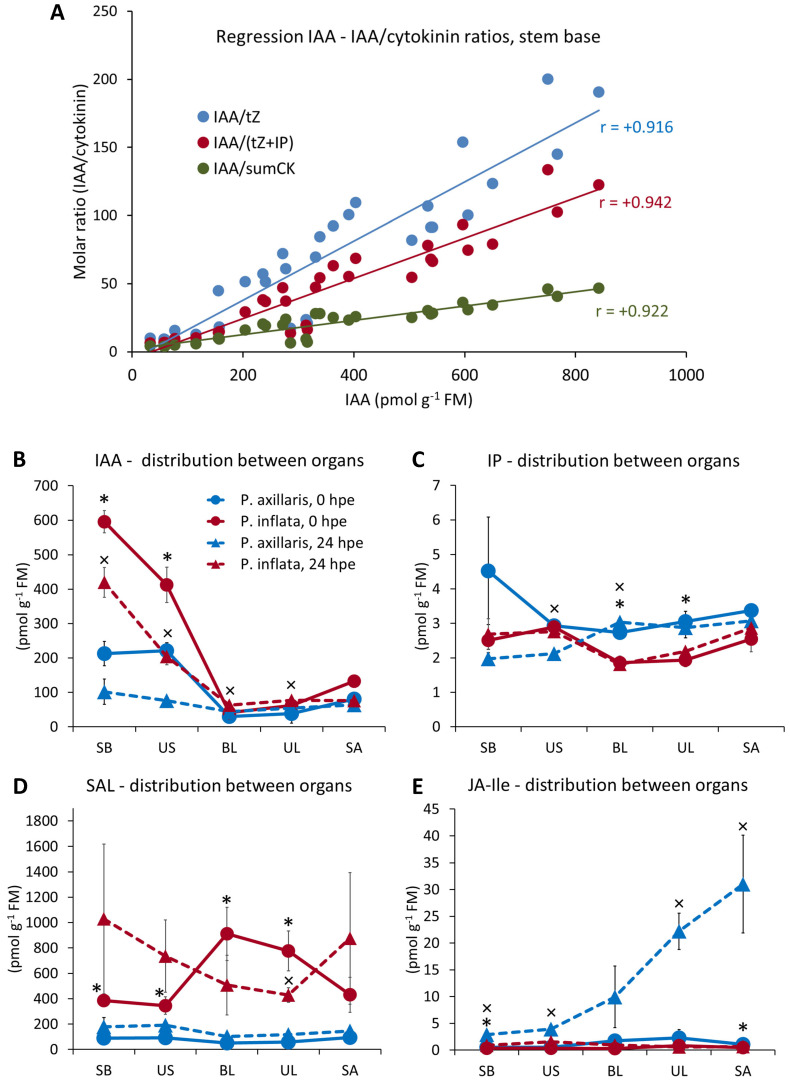
Linear regressions calculated between IAA in the stem base as independent variable and different IAA/cytokinin ratios as dependent variable, using combined data of *P. axillaris* and *P. inflata* from all time points (*n* = 30) **(A)**. Distribution of IAA **(B)**, IP **(C)**, SAL **(D)**, and JA-Ile **(E)** between the different cutting parts at 0 h and 24 hours post excision (hpe). Asterisks indicate significant differences between the two species at 0 hpe. Crosses indicate significant differences between the two species at 24 hpe. Each sample consisted of material from 10 or 16 cuttings for 24 or 0 hpe, respectively.

**Figure 9** shows hormone dynamics and IAA/cytokinin ratios in the stem base of *P. axillaris* and *P. inflata* after excision. In both species, JA peaked sharply at 0.5 hpe before dropping to baseline by 24 hpe, with *P. axillaris* showing nearly double the peak level of *P. inflata* (**Figure 9A**). OPDA followed a similar pattern, but *P. axillaris* reached over triple the peak level of *P. inflata* and maintained higher levels throughout (**Figure 9B**). JA-Ile, generally lower than JA, mirrored the pattern of JA but reached equally high peak levels in both species at 0.5 hpe (**Figure 9C**). *P. axillaris* showed slightly, but significantly, higher JA-Ile levels at 0, 2, and 24 hpe. IAA levels in the stem base were about three times higher in *P. inflata* than in *P. axillaris* at the time of cutting excision and remained elevated until root initiation at 72 hpe (**Figure 9D**). IAA rose until 2 hpe and then declined below initial levels. Trans zeatin (tZ), cis zeatin (cZ), isopentenyladenosine (IPR), and trans zeatin riboside (tZR) increased strongly in *P. inflata* after 24 hpe, reaching higher levels than *P. axillaris* (**Figures 9E–I**), while isopentenyladenine (IP) showed little variation (**Figure 9G**). IAA/cytokinin ratios mirrored IAA trends, peaking at 2 hpe and thereafter decreasing to a minimum at 72 hpe, then revealing only small interspecific differences (**Figures 9J–L**). IAA correlated strongly (*B* > 0.83) with these ratios (**Figure 10A**).

The analysis of the phytohormone-related transcriptome already indicated that excision of cuttings modified plant hormone metabolism at the whole-cutting level. Therefore, we also analyzed plant hormone concentrations at 0 and 24 hpe in the upper cutting parts in addition to the stem base. The results highlight a species-dependent distribution of certain plant hormones along the shoot axis that was additionally affected by the time after excision. In both species, the IAA levels at 0 hpe increased in basipetal direction along the shoot axis, showing the lowest levels in the leaves and an increase from the shoot apex toward the stem base (**Figure 10B**). However, this gradient was much stronger in *P. inflata* than in *P. axillaris*. Furthermore, the basipetal increase of IAA along the shoot axis in *P. inflata* remained at a high level until central root induction (24 hpe), whereas in *P. axillaris*, the gradient almost completely disappeared by 24 hpe (**Figure 10B**). At both time points, the gradients of IAA concentration between basal leaves or the upper leaves and the stem base were significantly higher in *P. inflata* compared to *P. axillaris* (**Supplementary Table S5**).

Concerning the other hormones, the most temporally stable difference between the two species in the upper cutting parts was a significantly lower level of IP in leaves for *P. inflata* compared to *P. axillaris* (**Figure 10C**). Trans-zeatin increased in the upper cutting sections between 0 and 24 hpe, and at 24 hpe, it only differed between the species in the upper stem, with lower levels in *P. inflata* (**Supplementary Table S6**). The concentrations of tZR were highest in the shoot apex and the upper stem, while *P. inflata* revealed significantly higher levels than *P. axillaris* in all stem sections at 0 hpe and in all cutting sections at 24 hpe (**Supplementary Table S6**). At 0 hpe, SAL concentrations in *P. inflata* showed the highest levels in the leaves and were higher than in *P. axillaris* in all cutting sections (**Figure 10D**). The SAL gradient between leaves and the stem base of *P. inflata* became inverted between 0 and 24 hpe. Nevertheless, *P. inflata* maintained higher SAL levels in the leaves than *P. axillaris*. JA-Ile was similarly low in all tissues of both species at 0 hpe but showed an apical increase along the shoot axis only in *P. axillaris* at 24 hpe (**Figure 10E**). Furthermore, *P. inflata* revealed lower JA levels in most tissues at 24 hpe than *P. axillaris* (**Supplementary Table S6**).

**Figure 11:**
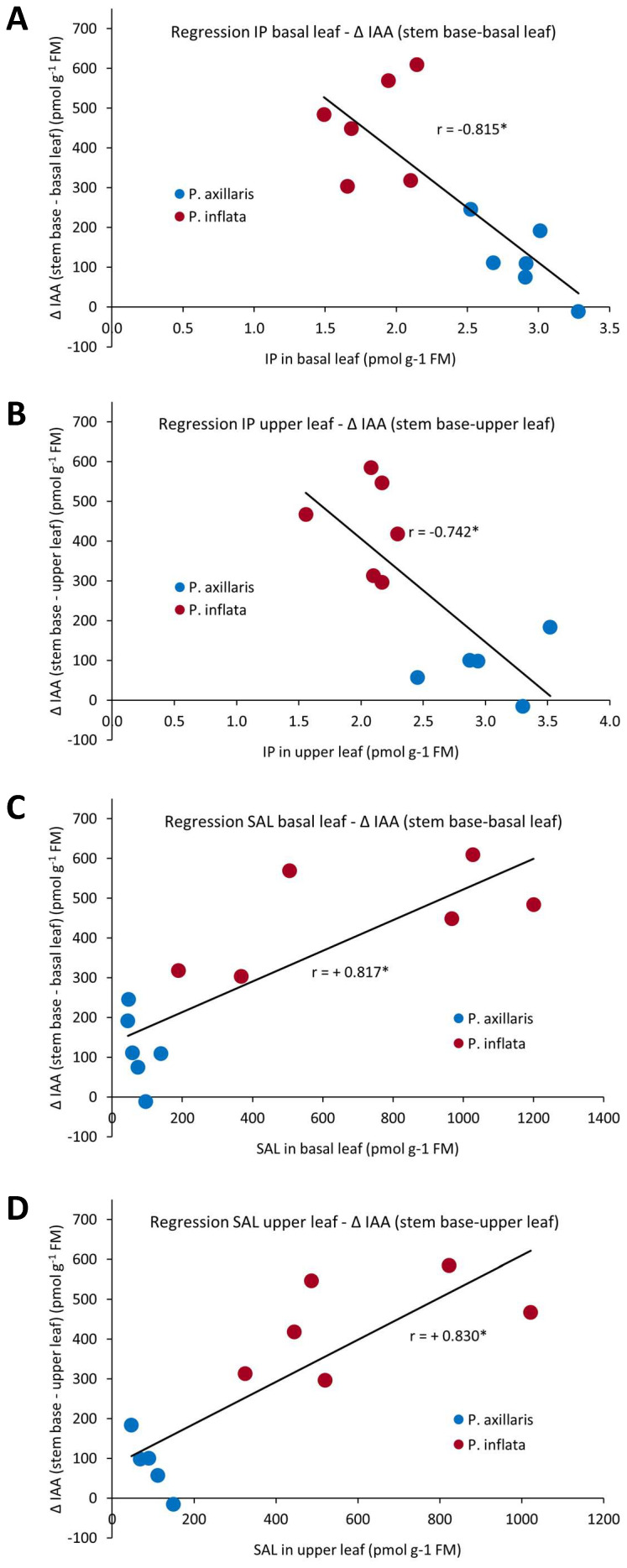
Linear regressions calculated between IP in the basal leaf **(A)**, IP in the upper leaf **(B)**, SAL in the basal leaf **(C)**, and SAL in the upper leaf **(D)** as the independent variables and the IAA concentration gradient between the respective leaf and the stem base as the dependent variable. Combined data of *P. axillaris* and *P. inflata* from 0 and 24 hours post excision (hpe) (*n* = 12). Each sample consisted of material from 10 or 16 cuttings for 24 or 0 hpe, respectively.

Correlations between leaf hormone levels and the IAA gradient to the stem base were analyzed (**Supplementary Table S7**). Stem-base IAA was unrelated to leaf IAA levels but strongly correlated with the leaf–stem gradient (*r* = 0.997), indicating that transport intensity rather than leaf auxin levels determined stem-base IAA. The IAA gradient was negatively correlated with leaf IP, JA, OPDA, and JA-Ile (**Figures 11A, B**, **Supplementary Table S7**) but positively correlated with leaf SAL (**Figures 11C, D**). However, for JA, OPDA, and JA-Ile, data distributions were uneven (**Supplementary Figure S3**).

### Response of adventitious rooting of *P. axillaris* and *P. inflata* to stem base-targeted IAA application and to blocking of PAT from the upper shoot

**Figure 12:**
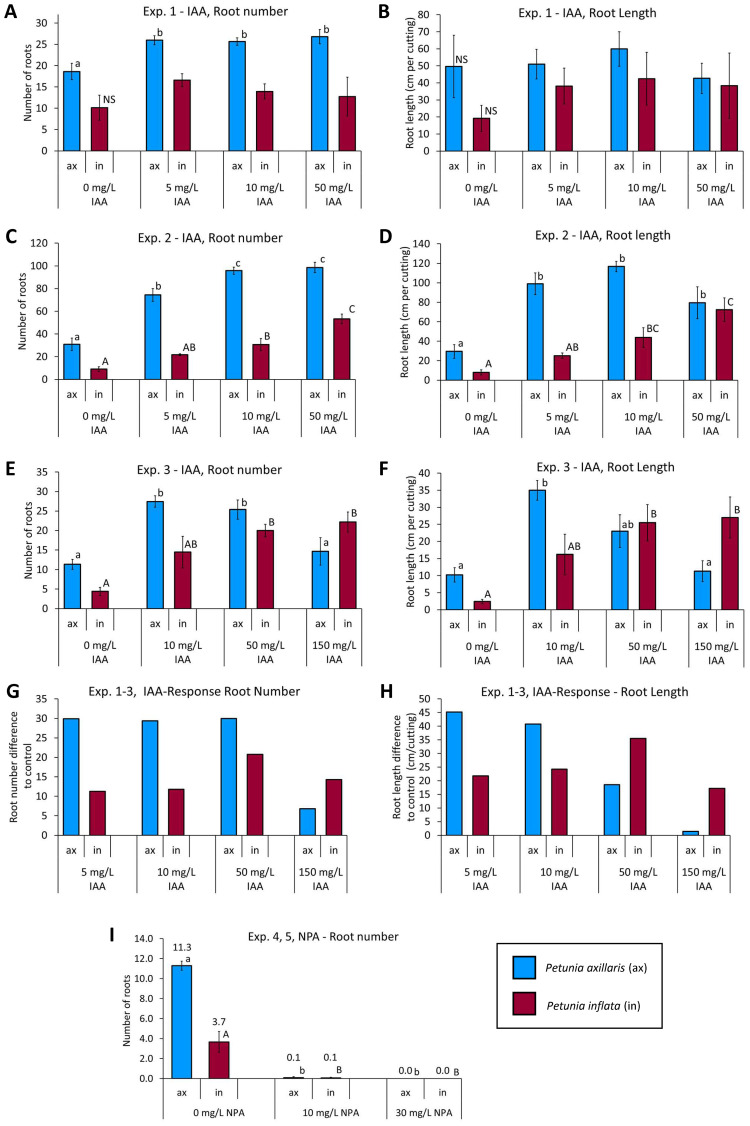
Rooting response of *P. axillaris* and *P. inflata* to applications of different dosages of IAA applied between 0 and 72 hours post excision **(A**–**H)** and to continuous lanolin-mediated applications of naphtylphatalamic acid (NPA) **(I)**. Number **(A**, **C**, **E)** and length **(B**, **D**, **F)** of formed adventitious roots per planted cutting in three independent experiments. Mean auxin response of root number **(G)** and root length **(H)** in terms of difference to the IAA-free control (0 mg L^−1^ IAA), calculated over the three experiments. Root number as affected by different dosages of NPA **(I)**. Columns and whiskers show the mean values ± SE. Numbers above the columns in **(I)** additionally show the mean values. Different upper- or lowercase letters indicate significant differences between the IAA and NPA dosages for *P. axillaris* or *P. inflata*, respectively (ANOVA, Tukey test, *p* < 0.05, *n* = 4 [Exp 1–3], *n* = 3 [Exp. 4, 5], each *n* consisting of 10 cuttings).

The transcriptome data, as well as the phytohormone levels in the stem base, indicated that *P. inflata*, which showed a higher rooting potential when compared with *P. axillaris*, benefits from the locally higher IAA levels that could evoke enhanced auxin signaling at the levels of *Aux/IAA*, *ARF*, *LBD*, and *PIN* gene transcripts during the induction phase, thereby promoting AR formation. If this were the case, AR formation in *P. axillaris* should benefit more from external IAA supply to the stem base than that in *P. inflata*. To test this hypothesis, in three replicative experiments, cuttings of both species were rooted under standard climate chamber conditions while being exposed to increasing concentrations of IAA for the first 72 h after excision. With the start of root initiation at 72 hpe, cuttings were replanted in perlite until rooting was assessed on day 16 after excision. In all experiments, cuttings of *P. inflata* produced fewer and shorter ARs compared with *P. axillaris* when no IAA was supplied (**Figures 12A–F**). However, root number and length of *P. axillaris* were significantly enhanced by the application of 5 mg L^−1^ IAA, whereas these parameters were not significantly affected by the same dosage in *P. inflata* (experiments 1 and 2, **Figures 12A–D**). Additionally, the application of 10 mg L^−1^ IAA resulted in a strong increase in root numbers and length in *P. axillaris* in all three experiments compared to the controls, whereas such an increase was nonsignificant (Exp 1, **Figures 12A, B**) or much smaller (**Figures 12C–F**) for *P. inflata*. By contrast, AR formation of *P. inflata* apparently benefited more from higher IAA dosages than *P. axillaris*. Thus, in experiments 2 and 3, the root number of *P. inflata* increased or remained at a similar level when IAA supply was increased from 10 to 50 mg L^−1^, respectively, whereas the root number of *P. axillaris* was maintained or even slightly decreased (**Figures 12C, E**). Further increase of IAA from 50 to 150 mg L^−1^ decreased the root number of *P. axillaris* but had no negative effect on *P. inflata* (**Figure 12E**). Increasing IAA supply above 10 mg L^−1^ reduced root length of *P. axillaris* in all three experiments, although not significant at the individual experimental level, while root length of *P. inflata* remained at the 10 mg L^−1^ level or even slightly increased with the 50 mg L^−1^ dosage in experiment 2 (**Figures 12B, D, F**).

**Figures 12G, H** show that the interspecific difference in auxin response depended on auxin concentration, depicting the mean absolute differences in root number and length at various IAA levels relative to the IAA-free control across three experiments. *P. axillaris* showed a higher increase in root number and length than *P. inflata* up to a dosage of 10 mg L^−1^ IAA. With 50 mg L^−1^ IAA, the increase in root number was only slightly higher in *P. axillaris* than in *P. inflata*, whereas the increase in root length was already lower in *P. axillari*s. With 150 mg L^−1^ IAA, *P. axillaris* showed a smaller increase in root number and length than *P. inflata*.

The exclusive upregulation of auxin transporter genes in *P. inflata*, together with a stronger IAA gradient between the leaves and the stem base compared to *P. axillaris*, supports the conclusion that the cutting leaves in *Petunia* are important auxin source organs, from which IAA is transported to the stem base, where it can induce the formation of ARs. To analyze the contribution of PAT-controlled auxin influx from cutting parts above the basal stem, in particular the leaves, into the stem base, two experiments were conducted with *P. inflata* and *P. axillaris*, respectively, using the PAT blocker NPA. Lanolin rings containing NPA at two concentrations or pure lanolin were placed around the bases of the two basal leaves and the stem immediately above the two basal leaves to interrupt, or not interrupt, the PAT-derived auxin flow from the upper shoot, including all leaves, to the stem base (**Figure 1B**). In both species, NPA application at 10 mg L^−1^ lanolin and 30 mg L^−1^ lanolin almost completely and completely inhibited AR formation, respectively (**Figure 12I**). The results document that the PAT-dependent basipetal transport of IAA from the upper shoot, including the leaves, to the stem base is a bottleneck of adventitious rooting in both species.

## Discussion

### *P. inflata* has a higher rooting capacity than *P. axillaris*, but the realization of rooting also depends on donor plant factors

Both species rooted efficiently and formed adventitious roots rapidly without exogenous auxin, but *P. inflata* generally showed higher rooting capacity (**Figure 2**). In some experiments, however, *P. axillaris* exhibited stronger rooting (**Figures 2**, **12**). As donor plants were cultivated under variable greenhouse conditions, while rooting always occurred under identical climate chamber conditions, these findings suggest that rooting is genetically controlled but also influenced by the physiological state of the donor plants. Rooting of *P. hybrida* Mitchell cuttings, for instance, depends on nitrogen supply to donor plants without affecting auxin homeostasis (Zerche et al., 2016; Yang et al., 2019). Likewise, photosynthesis of cuttings, influencing carbohydrate supply for root growth (Rapaka et al., 2005), is modified by donor plant light conditions (Klopotek et al., 2012). Since nitrogen nutrition was optimized based on repeated substrate analysis in this study, variations in light, temperature, and plant age likely affected rooting, with *P. inflata* apparently less resilient than *P. axillaris*. Nevertheless, the data as a whole indicate a higher rooting capacity of *P. inflata*, since the minimum level of rooting that could be reached across all experiments was higher in this species (**Figures 2**, **12**).

### Both species reveal an early activation of the jasmonic acid pathway in the stem base during the root induction phase, which is further linked to the ERF family

The phytohormone-targeted microarray proved to be a reliable and valuable tool for monitoring hormone-related transcriptomes in both *Petunia* species (**Supplementary Figures S1**, **S2**). One of the earliest responses in the stem base after cutting excision was the upregulation of genes controlling JA biosynthesis and signaling at 0.5 hpe, followed by genes for JA-Ile biosynthesis at 2 hpe (**Figures 3C–F**). This was accompanied by a marked increase in JA and JA-Ile levels in the same tissue, peaking at 0.5 hpe (**Figures 9A, C**). While JA levels peaked higher in *P. axillaris*, JA-Ile reached similar maxima in both species. Considering that JAZ genes are among the earliest JA-induced genes (Wasternack and Song, 2017), the stronger and more persistent JAZ activation in the stem base (**Figure 3E**) and upper stem (**Figure 8C**) of *P. inflata* indicates enhanced JA signaling compared with *P. axillaris*. Upregulation of JA biosynthesis and signaling is a typical response to wounding (Wasternack and Song, 2017). A similarly early, transient JA accumulation in the stem base was reported for *P. hybrida* and pea cuttings (Ahkami et al., 2009; Rasmussen et al., 2015). Although JA can inhibit etiolation-induced AR formation in *Arabidopsis* hypocotyls, involving changes in auxin and cytokinin homeostasis and signaling (Gutierrez et al., 2012; Lakehal et al., 2019; Dob et al., 2021), accumulating evidence supports its positive regulatory role in cuttings. Suppression of wound-induced JA synthesis by AOC silencing reduced AR formation in *P. hybrida* cuttings (Lischweski et al., 2015), whereas short-term JA application to pea cuttings promoted rooting (Rasmussen et al., 2015). These findings support the hypothesis that early activation of the JA pathway contributes to AR formation in *P. axillaris* and *P. inflata* and may also partially underlie the higher rooting capacity of *P. inflata*. The stimulation of the JA pathway extended beyond the stem base, reaching upper leaves and the shoot apex at 24 hpe (**Figures 8A, B**, **10E**, **Supplementary Table S6**), suggesting JA involvement in systemic processes supporting AR formation.

*ERF* genes showed frequent and strong upregulation, starting in the stem base at 0.5 hpe (**Figure 7A**) and, in specific cases, also in upper cutting sections at 24 hpe (**Figure 7B**). In *Arabidopsis*, depending on the specific *ERF* gene, expression can be regulated by ethylene as well as by other plant hormones such as JA, ABA, and SAL (Heyman et al., 2018). *ERF* genes have various functions, including wound response, tissue repair, stress tolerance, pathogen resistance, and plant development (Heyman et al., 2018; Wu et al., 2022). In this study, both species showed local upregulation in the stem base of *ERF1*, *ERF2*, *ERF4*, *ERF5*, *ERF13*, *ERF17*, and *ERF25*. In *Arabidopsis*, *ERF1* integrates ethylene and JA signals (Lorenzo et al., 2003) and regulates primary and lateral root development involving upregulation of auxin transporters (Mao et al., 2016; Zhao et al., 2023). *ERF2* controls root growth in rice and affects responses to ethylene, ABA, auxin, and cytokinins (Xiao et al., 2016). Genetically engineered upregulation of auxin biosynthesis in apple rootstocks enhanced *ERF5* expression in both rootstocks and scions (Zhai et al., 2021), and overexpression of *ERF5* in *Dendrobium* orchid promoted regeneration of protocorm-like bodies (Zeng et al., 2023). In this study, *P. axillaris* showed strong upregulation of *ERF114* in the stem base from 24 hpe onward (**Figure 7A**) and in the upper stem at 24 hpe (**Figure 7B**). *P. inflata* exhibited local upregulation of *ERF113*, a close homolog of *ERF114*, between 2 and 72 hpe (**Figure 7A**) and in all upper cutting parts except the shoot apex at 24 hpe (**Figure 7B**). In *Arabidopsis*, *ERF113* is responsive to JA, SAL, ABA, and ethylene (Heyman et al., 2018). Both *ERF113* and *ERF114* are rapidly induced by wounding (Ikeuchi et al., 2017) and play roles in etiolation-induced AR formation in hypocotyls of *Arabidopsis* seedlings, where JA acts as a negative regulator (Lakehal et al., 2020). These results indicate that early upregulation of distinct *ERF* genes, such as *ERF1*, *ERF2*, *ERF5*, and particularly *ERF113*/*ERF114*, contributes to AR induction in *P. axillaris* and *P. inflata*, possibly acting downstream of JA and ethylene and upstream of auxin.

### Early local upregulation of ILR/ILL genes and PINOID-mediated rearrangement of intracellular auxin distribution seem to contribute to the auxin homeostasis in the stem base of *P. inflata* and *P. axillaris*

The highest number of DEGs occurred in the auxin category (**Figures 3A, B**). Expression data from the stem base showed predominant downregulation of genes controlling auxin biosynthesis, particularly *YUCCA* genes (**Figures 4A, B**), contrasting sharply with the upregulation of several *YUCCA* genes in *Arabidopsis* leaf explants during AR formation (Chen et al., 2016). Both species, however, showed rapid and strong upregulation of *ILR1-like2* and *ILR1-like6*, and *P. axillaris* additionally upregulated *ILR1-like4* (**Figures 4A, B**, 5B). *ILR/ILL* genes encode auxin-conjugate hydrolases that have been extensively characterized in *Arabidopsis*. In this plant, specific genes exhibit distinct but overlapping substrate specificities for conjugates between IAA or IBA and individual amino acids (Smolko et al., 2018), and hydrolases of the ILR1-like family identified regulate auxin homeostasis in the endoplasmic reticulum (ER) (Carranza et al., 2016). *ILR* genes can be induced by wounding and JA. In this context*, ILR1*, *ILL6*, and *IAR3*, members of the *ILR1*-like family (Campanella et al., 2003), were upregulated in *Arabidopsis* by wounding and JA and not only controlled auxin-conjugate hydrolysis but also reduced JA signaling via hydrolysis of JA-Ile (Zhang et al., 2016b). These findings suggest that early upregulation of *ILR1-like2*, ILR1-like4, and *ILR1-like6* contributes to the increase in IAA at 2 hpe and possibly to the simultaneous decrease in JA-Ile in *P. axillaris* and *P. inflata* (**Figures 9C, D**), thereby influencing AR formation.

PINOID protein serine/threonine kinases and their close homologs WAG1 and WAG2 phosphorylate PIN proteins, directing them to the apical cell side, whereas PP2A phosphatase antagonistically dephosphorylates PINs, targeting them basally (Friml et al., 2004; Michniewicz et al., 2007; Adamowski and Friml, 2015; Armengot et al., 2016). In this study, one gene of the *PINOID*/*WAG1*/*WAG2* family was downregulated at 0.5 hpe in each species (**Figure 4C**; Peaxi162Scf00333g00017.1, Peinf101Scf00423g01001.1 in **Supplementary Table S3**). Both genes share high identity with the *PINOID* gene A4A49_10797 (protein ID AMO02498.1) of *Nicotiana attenuata*. Overexpression of a *PINOID* gene in rice delayed crown root formation, a type of AR formed on intact monocot seedlings (Morita and Kyozuka, 2007). In *Arabidopsis*, AR formation in the hypocotyl of pre−etiolated flooded seedlings was strongly inhibited in the triple mutant *pid14 wag1 wag2*, even when external auxin was applied, indicating that proper PIN phosphorylation by these kinases is essential for AR establishment (Da Costa et al., 2020). These findings suggest that early downregulation of the A4A49_10797 homolog in the stem bases of *P. axillaris* and *P. inflata* may enhance basipetal IAA transport, directing auxin toward the founder cells initiating AR formation.

The preferential downregulation of *CCD*s and *D27*, which control SL biosynthesis, observed in *P. axillaris* and *P. inflata*, is consistent with similar findings in *P. hybrida* Mitchell (Bombarely et al., 2016). These results support the hypothesis that SL downregulation contributes to AR formation in both species, possibly by reducing the inhibitory influence of SL on PAT and AR initiation, as reported in *Arabidopsis* and pea (Rasmussen et al., 2012; Shinohara et al., 2013; Koltai, 2014).

### Rooting of *P. inflata* benefits from a higher auxin accumulation in the stem base that determines a higher auxin/cytokinin ratio and promotes the auxin signal transduction during AR induction

In both species, IAA increased after cutting excision until 2 hpe and then decreased to below the initial level, with consistently higher concentrations in *P. inflata* (**Figure 9D**). Changes in auxin transporter expression in the stem base were mainly detected at 24 hpe, with *PIN*/*PIN-like* genes more frequently upregulated in *P. inflata* (**Figures 4C, D**, **5C**). This suggests that transporter gene regulation in the stem base was a consequence of elevated IAA levels rather than their cause. In hypocotyls of pre−etiolated *Arabidopsis*, auxin−responsive rearrangement of transporter expression has been shown to drive cell reprogramming and subsequent differentiation during adventitious rooting (Della Rovere et al., 2013). Similarly, auxin transporters displayed auxin−responsive, phase−specific expression profiles in the stem bases of tomato and olive cuttings (Guan et al., 2019; Velada et al., 2020). Both *Petunia* species showed upregulation of *PIN6* at 24 hpe during AR induction and of *PIN5* at 72 hpe, corresponding to the expected onset of AR initiation (**Figure 5C**). In *Arabidopsis*, *PIN5* encodes a functional auxin transporter localized to the ER and is required for auxin−mediated development (Mravec et al., 2009). PIN6 localizes to both the ER and plasma membrane, mediating auxin transport across the plasmalemma and regulating intracellular auxin homeostasis (Cazzonelli et al., 2013; Simon et al., 2016). Overexpression of *PIN6* promoted AR formation in intact and de−rooted *Arabidopsis* seedlings (Cazzonelli et al., 2013; Simon et al., 2016). Only *P. inflata* exhibited upregulation of *PIN-like1* and/or *PIN-like3* genes, with one *PIN-like3* gene already induced at 24 hpe (**Figure 5C**). These findings highlight *PIN6* as a key auxin transporter for AR induction in *Petunia*, supported in *P. inflata* by *PIN-like3*.

Consistent with the higher maximum IAA level in the stem base at 2 hpe (**Figure 9D**) and more frequent upregulation of auxin transporters thereafter (**Figure 5D**), *P. inflata* also showed stronger upregulation of *Aux/IAA* and *ARF* genes controlling auxin signaling (**Figures 6A, B**). *Aux/IAA* proteins act as auxin repressors of *ARF* proteins, which are positive regulators directly controlling transcription of auxin-responsive genes such as *LBD* or *GH3* (Leyser, 2018). Auxin-induced degradation of specific *Aux/IAA* proteins subsequently enhanced transcription of their own encoding genes (Krogan and Berleth, 2015). Thus, the more frequent upregulation of *Aux/IAA* genes in *P. inflata* (**Figure 4E**) likely reflects stronger auxin perception, consistent with its higher IAA concentration (**Figure 9D**). In this study, one and two genes homologous to *ARF11* were strongly upregulated between 2 and 72 hpe in the stem base of *P. axillaris* and between 0.5 and 72 hpe in *P. inflata*, respectively (**Figure 6B**). In *Arabidopsis*, mutation of *ARF11* reduced lateral root number and length (Yamauchi et al., 2024). One homolog of *ARF9* was downregulated between 2 and 72 hpe in *P. axillaris* and at 2 hpe in *P. inflata*. In transgenic tomato plants, increasing or decreasing *ARF9* transcript levels suppressed or enhanced cell division during early fruit development, respectively, demonstrating an inhibitory function of *ARF9* in cell division (de Jong et al., 2015). A similar inhibitory role may apply to *ARF9* during early cell division events of AR formation in *P. inflata* and *P. axillaris*, suggesting that upregulation of *ARF11* combined with downregulation of *ARF9* supports AR formation in both species.

Genes of the *LBD* family, which are regulated by auxin and other phytohormones such as jasmonate and ethylene (Majer and Hochholdinger, 2011; Rong et al., 2024), responded strongly to cutting excision in both *Petunia* species. More *LBD* genes were upregulated in *P. inflata* than in *P. axillaris*, with differences apparent from 2 hpe onward (**Figure 6C**). In both species, one homolog of *LBD41* was upregulated from 0.5 hpe throughout the sampling period until 72 hpe (**Figure 6C**). At 24 hpe and 72 hpe, *LBD41* upregulation was stronger in *P. inflata* (*M*-values 5.27 and 7.03) than in *P. axillaris* (*M*-values 3.54 and 5.88) (**Supplementary Table S3**). Consistent with our findings, *LBD41* was also induced in the stem base of de-rooted mung bean seedlings at 6 and 24 hpe, and hydrogen peroxide stimulation of AR formation further enhanced its expression (Li et al., 2017). In both *Petunia* species, homologs of *LBD1* were upregulated from 2 hpe onward (**Figure 6C**). While the role of *LBD1* in adventitious rooting remains unclear, it has been linked to secondary growth and cambium differentiation (Rong et al., 2024). In rice, *LBD1–8* acts downstream of auxin signaling and is highly expressed in the cortex and lateral root primordia, with lateral root formation depending on its expression (Yamauchi et al., 2019). In both *Petunia* species, one homolog of *LBD16* was upregulated during the induction phase (24 hpe) and at AR initiation (72 hpe) (**Figure 6C**). Similarly, *LBD16* is induced in *Arabidopsis* leaf explants during early AR induction, regulating AR organogenesis in an auxin-dependent manner (Liu et al., 2014, 2018). Furthermore, overexpression of an apple homolog of *LBD16* in tomato increased the number of ARs (Wang et al., 2023). Overall, the data indicate more frequent and stronger *LBD* gene upregulation in the stem base of *P. inflata* compared with *P. axillaris*, consistent with its higher IAA levels, stronger auxin signaling activation, and greater rooting capacity. In both species, *LBD1*, *LBD16*, and *LBD41* appear to play major roles in excision-induced AR formation, likely acting downstream of auxin and possibly other phytohormones.

In addition to the early upregulation of homologs of *JAR1* (**Figures 3E, F**), which is *GH3.11* in *Arabidopsis* (Wasternack and Song, 2017), both *Petunia* species showed preferential upregulation of other *GH3* genes in the stem base (**Figure 4G**). At 2 and 24 hpe, more *GH3* genes were upregulated in *P. inflata* than in *P. axillaris*. Many GH3 proteins act as IAA−amidosynthetases and/or JA−amidosynthetases (Jez, 2022), so *GH3* upregulation may have contributed to the reduction of free IAA and JA toward AR initiation (72 hpe in **Figures 9A, D**). Considering the inhibitory roles of high JA and IAA levels during AR initiation (Da Costa et al., 2013), *GH3* induction may thus facilitate final AR formation in *P. axillaris* and *P. inflata*.

Differentially expressed *SAUR* genes (**Figure 4G**) are also likely to play crucial roles in AR formation in both species. SAUR proteins are transcriptionally induced by auxin in various plant species and are involved in hormone−mediated development (Ren and Gray, 2015), although auxin−induced SAUR repression has also been observed (Paponov et al., 2009). In shoots, specific SAURs control cell expansion by targeting PP2C.D phosphatases (Spartz et al., 2014; Ren and Gray, 2015; Fendrych et al., 2016; Ren et al., 2018). Recent studies in *Arabidopsis*, cucumber, and sweet potato have shown that specific SAUR proteins can also respond to jasmonate, can promote lateral root and AR formation by stimulating cell expansion, and, in some cases, act upstream of auxin by increasing auxin levels (Yin et al., 2020; Luan et al., 2023; Zhou et al., 2024).

The higher rooting capacity of *P. inflata* versus *P. axillaris* can be explained by the higher IAA levels, the higher IAA/cytokinin ratio, the associated stronger auxin signaling at the levels of *Aux/IAA* and *ARF* transcription, and the stronger downstream response, as reflected by the higher *PIN/PIN-like*, *GH3*, and *LBD* expression. The idea that the lower endogenous IAA level is a key factor for the lower rooting capacity of *P. axillaris* is supported by the finding that rooting of this species responded more positively to low IAA dosages of 5 to 10 mg L^−1^ IAA during root induction than did *P. inflata* (**Figure 12**). However, the finding that rooting of *P. inflata* was stronger enhanced by high IAA dosages of 50 mg and 150 mg L^−1^, than that of *P. axillaris* further reveals a higher maximum auxin response capacity for *P. inflata*. The increase in various cytokinins in the stem base of *P. inflata* between 24 and 72 hpe suggests a role in establishing new cell clusters—the earliest signs of root initiation in *Petunia* (Ahkami et al., 2009). In this context, cytokinins are well-known promoters of cell division within the quiescent center of *Arabidopsis* roots (Schaller et al., 2014).

### Rooting of both species is dependent on leaf-derived, PAT-controlled auxin supply, which determines a leaf-stem base auxin gradient, while *P. inflata* obviously benefits from higher transcription of *PIN* and *PIN-like* transporters in the leaves

NPA inhibits auxin efflux from plant cells, likely by affecting PIN- and ABCB-mediated auxin transport (Teale and Palme, 2018). Application of 30 mg L^−1^ NPA in lanolin around basal leaf bases and the upper stem of *Petunia* cuttings (**Figure 1**) completely prevented rooting in both species (**Figure 12I**). This demonstrates that auxin influx from the upper cutting parts, including leaves, into the stem base is essential for AR formation of both species. *P. inflata* showed a steeper IAA gradient between leaves, upper stem, and stem base (**Figure 10B**, **Supplementary Table S5**), associated with exclusive upregulation of *PIN5* and *PIN*-*like* genes in leaves (**Figure 5D**). Auxin transporters at the ER, such as PIN5 and PIN-like, have been suggested to reduce auxin availability for signaling and export from the cell by sequestration of IAA into the ER (reviewed in Ung, 2024). However, in a recent study by Zheng et al. (2025), virus-induced gene silencing of *PIN-like2*, which is expressed in the ER, decreased the expression of *Aux/IAA* and *GH3* genes and caused significant reductions in the number and length of formed roots. Further molecular assays in the same study demonstrated that one member of the TGA bZIP transcription factor family, TGA7, directly binds the promoter of *PIN-like2* and stimulates its transcription (Zheng et al., 2025). TGA factors are key components in SAL signaling activated by SAL (Seyfferth and Tsuda, 2014).

### Leaf SAL and cytokinin levels may control the leaf-derived auxin supply to the stem base of cuttings

Negative correlations were found between leaf IP levels and the leaf–stem base IAA gradient (**Figures 11A, B**). Cytokinins regulate *PIN* gene transcription and PIN protein trafficking within cells, enhancing auxin transport in shoots related to shoot branching (Marhavy et al., 2014; Simásková et al., 2015; Waldie and Leyser, 2018). *P. inflata* cuttings showed higher SAL levels in leaves compared to *P. axillaris* (**Figure 10D**), which were positively correlated with the IAA gradient between leaves and stem base (**Figures 11C, D**). SAL influences auxin transport and adventitious rooting, though its roles are not fully understood. SAL application increased transcripts of four *PIN* genes in cotton shoots (He et al., 2017) but inhibited rootward [^3^H-IAA] transport and reduced *PIN* activity in *Arabidopsis* roots via hyperphosphorylation (Tan et al., 2020). Continuous SAL supply enhanced AR formation in cucumber hypocotyl cuttings (Dong et al., 2020). However, genetic reduction of SAL biosynthesis in *Arabidopsis* increased rooting percentage, while SAL treatment reduced it (Tran et al., 2023). Considering that these findings are limited to other plant species, lower IP and higher SAL levels in *P. inflata* may promote auxin export from leaves by unknown mechanisms, possibly involving activation of *PIN-like* genes via TGA transcription factors, as previously discussed.

## Conclusive model

**Figure 13:**
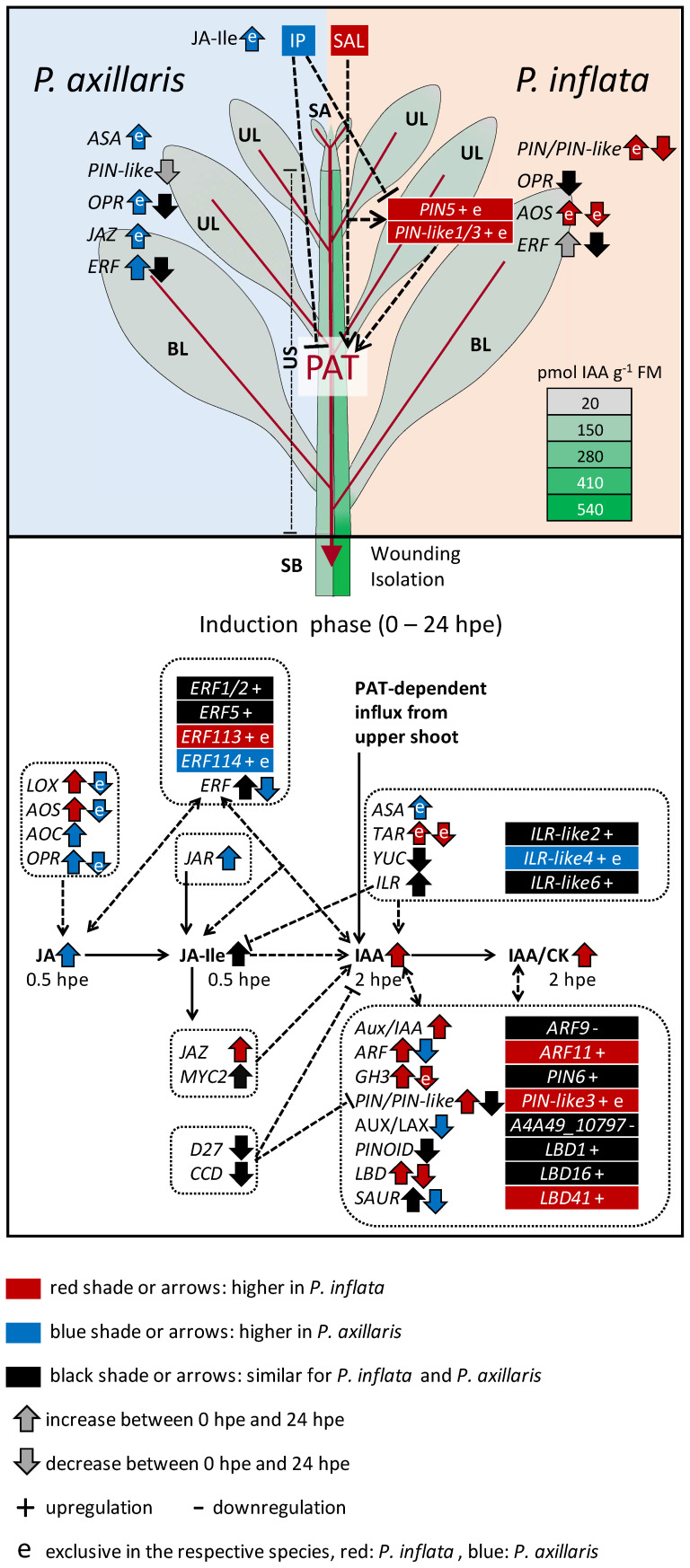
Conceptual model of hormone action in cuttings of *P. axillaris* and *P. inflata* during AR induction (0 until 24 hpe), highlighting important plant hormones, gene families, and genes. In the upper part, the blue- and reddish-shaded halves represent the upper cutting parts of *P. axillaris* and *P. inflata*, respectively. There, the increase of JA-Ile between 0 and 24 hpe (applies only to *P. axillaris*), the difference in IP and SAL levels between the two species, and the regulation of genes in the leaves of the two species are illustrated. The distribution of IAA (mean level calculated between 0 and 24hpe) among the five cutting sections of the two species is indicated by the intensity of green coloring in the separated right and left halves. In the lower part, the dynamics of hormone homeostasis and the regulation of important genes are illustrated for both species. Here, different responses of *P. axillaris* versus *P. inflata* or common responses of both species are indicated by blue, red, or black shading and the same coloring of the arrows. Red lines and arrows represent the PAT stream in the cutting. Solid and broken lines and arrows indicate established and putative interrelationships, respectively. Further explanation is given in the legend.

The data highlight a coordinated activation of the hormonal machinery in the stem base of the two *Petunia* species during AR induction. This process begins with the activation of JA and ERF transcription factors, followed by auxin, which depends strongly on auxin influx from the upper cutting sections. A conceptual model of these relationships is shown in **Figure 13**. Wounding triggers early JA accumulation in the stem base, reaching higher levels in *P. axillaris* and coinciding with enhanced expression of JA biosynthetic genes—*OPR* and *AOC* in *P. axillaris*, and *LOX* and *AOS* in *P. inflata*. Although *JAR* upregulation is higher in *P. axillaris*, JA-Ile peaks at similar levels in both species and is perceived via the COI1–JAZ co-receptor complex, reflected by elevated *MYC2* and *JAZ* expression. The stronger *JAZ* upregulation in *P. inflata* indicates a more pronounced JA response. Several wound-responsive *ERF* genes (*ERF1*, *ERF2*, *ERF5*) are induced in both species, while *ERF113* and *ERF114* are induced in a species-specific manner. The ERF and jasmonate pathways likely interact with the auxin pathway. Jasmonate and ERF activation also occur in the upper cutting parts of *P. axillaris*, as shown at 24 hpe by transcriptomic and JA-Ile data. During AR induction, PAT directs IAA basipetally from the upper sections to the stem base, with leaves likely serving as auxin sources. Downregulation of *CCDs* and *D27* may promote auxin transport and IAA accumulation by reducing inhibitory SLs. Exclusive upregulation of *PIN5* and *PIN-like1/3* in *P. inflata* leaves likely enhances auxin export, resulting in stronger basipetal IAA transport and a steeper IAA gradient, controlled by higher SAL and lower IP levels. Consequently, *P. inflata* shows increased IAA accumulation and a higher IAA/cytokinin ratio, favoring AR induction. This elevated ratio activates auxin signaling genes regulating IAA perception, signal transduction, and cellular response, leading to feedback changes in IAA distribution. Downregulation of *PINOID* (A4A49_10797) may favor basal PIN protein accumulation. Stronger induction of *Aux/IAA*, *ARF*, *GH3*, *PIN/PIN-like*, and *LBD* genes in *P. inflata* likely contributes to its higher rooting capacity compared to *P. axillaris*. Local upregulation of *ARF11* and *PIN6*, downregulation of *ARF9*, and exclusive induction of *PIN-like3* in *P. inflata* suggest key roles for these genes in AR induction. Upregulation of *LBD1*, *LBD16*, and *LBD41* in both species supports their function in auxin-dependent AR formation. Specific *ILR-like* genes may regulate the JA-Ile decrease after 0.5 hpe via deconjugation, facilitating AR initiation.

These findings enhance the understanding of hormonal regulation of adventitious rooting and provide a basis for the functional analysis of candidate genes using *Agrobacterium*-mediated transformation and CRISPR/Cas mutagenesis (Zhang et al., 2016a).

## Data Availability

The original data has now been published in GEO under the accession number: GSE317986.https://www.ncbi.nlm.nih.gov/geo/query/acc.cgi?acc=GSE317986.

## Glossary

ABCB: B family of membrane-bound ATP-binding cassette (ABC) transporters
AOC: allene oxide cyclase
AOS: allene oxide synthase
AR: adventitious root
ARF: auxin response factor
ASA: anthranilate synthase
AUX: auxin-resistant
Aux/IAA: auxin indole-3-acetic protein
BL: basal leaves
cZ: *cis* zeatin
dpe: days post excision
ET: ethylene
ERF: ethylene response factor
FMO-GC-OX-like: flavin-monooxygenase glucosinolate-*S*-oxygenase-like
GH3: Gretchen Hagen 3
GRAS: GRAS, GAI, RGA, and SCR like
hpe: hours post excision
IAA: indole-3-acetic acid
ILL: IAA-leucine resistant-like
ILR: IAA-leucine resistant
IP: isopentenyladenine
IPR: isopentenyladenosine
JA: jasmonic acid
JA-Ile: jasmonoyl-isoleucine
JAR: JA-amino acid synthetase
JAZ: jasmonate ZIM domain
LAX: like AUX
LBD: lateral organ boundaries domain
LOX: lipoxygenase
MYC2: myelocytomatosis 2
NPA: 1-*N*-naphthylphthalamic acid
NINJA: novel interactor of JAZ
OPR: 12-oxophytodienoic acid reductase
OPDA: 12-oxophytodienoic acid
PAT: polar auxin transport
PIN: PIN-formed
PINOID: protein serine/threonine kinase PINOID
SAL: salicylic acid
SAUR: small auxin up RNA
SA: shoot apex
SB: stem base
SL: strigolactones
TAR: tryptophan amino transferase
tZ: trans zeatin
tZR: trans zeatinriboside
UL: upper leaves
US: upper stem
WOX: WUSCHEL-related homeobox
YUCCA: YUCCA family of flavin monooxygenase
WAG1/WAG2: protein serine/threonine kinases WAG1 and WAG2
