# Supplementary material for: Transcriptomic and hormonal dynamics in relation to adventitious rooting of two parental Petunia species highlight a coordinated activation of the jasmonate and auxin pathways and an important role of upper-shoot-derived auxin influx
Source: Front Plant Sci. 2026 Feb 6;16:1707238. doi: 10.3389/fpls.2025.1707238 (PMC12920520; doi:10.3389/fpls.2025.1707238)
Supplement: Supplementary file 1 [file DataSheet1.pdf]

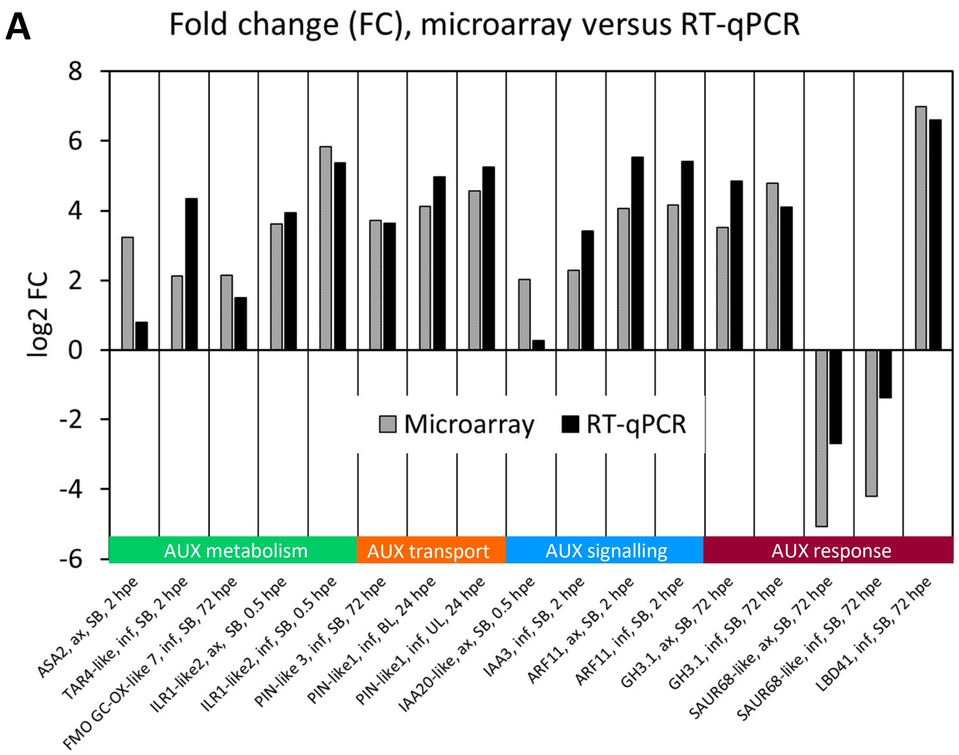

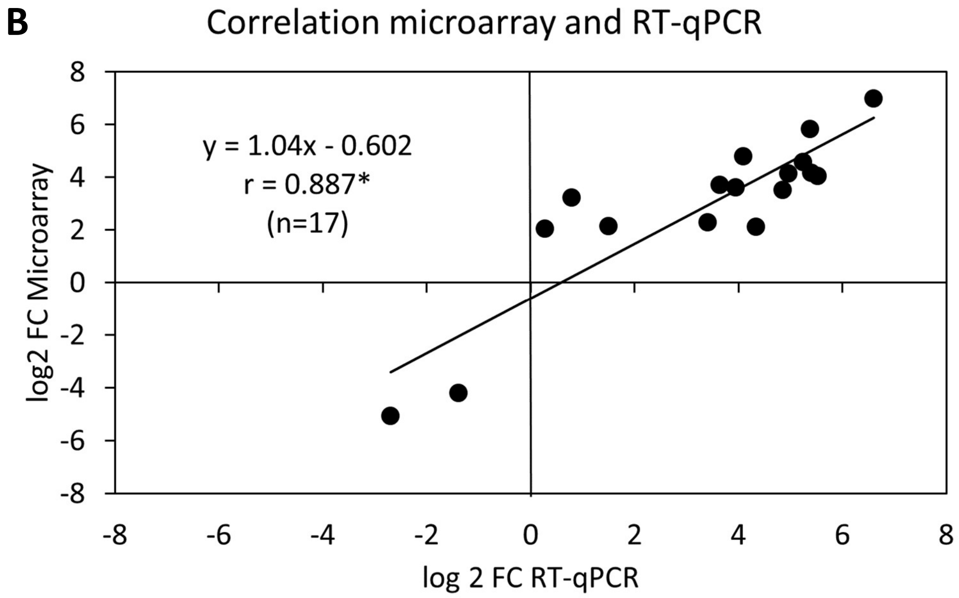

Supplementary Figure S1. Comparison of microarray and RT-qPCR results of transcriptome analysis of 17 selected auxin-related genes of different categories analyzed at selected hours post excision (hpe) in the stem base (SB), upper leaves (UL) or basal leaves (BL) of *P. axillaris* and *P. inflata*. Direct comparison of fold changes at a logarithmic scale (A) and correlation between fold changes analyzed by RT-qPCR and microarray (B).
