# Supplementary material for: Transcriptomic and hormonal dynamics in relation to adventitious rooting of two parental Petunia species highlight a coordinated activation of the jasmonate and auxin pathways and an important role of upper-shoot-derived auxin influx
Source: Front Plant Sci. 2026 Feb 6;16:1707238. doi: 10.3389/fpls.2025.1707238 (PMC12920520; doi:10.3389/fpls.2025.1707238)
Supplement: Supplementary file 2 [file DataSheet2.pdf]

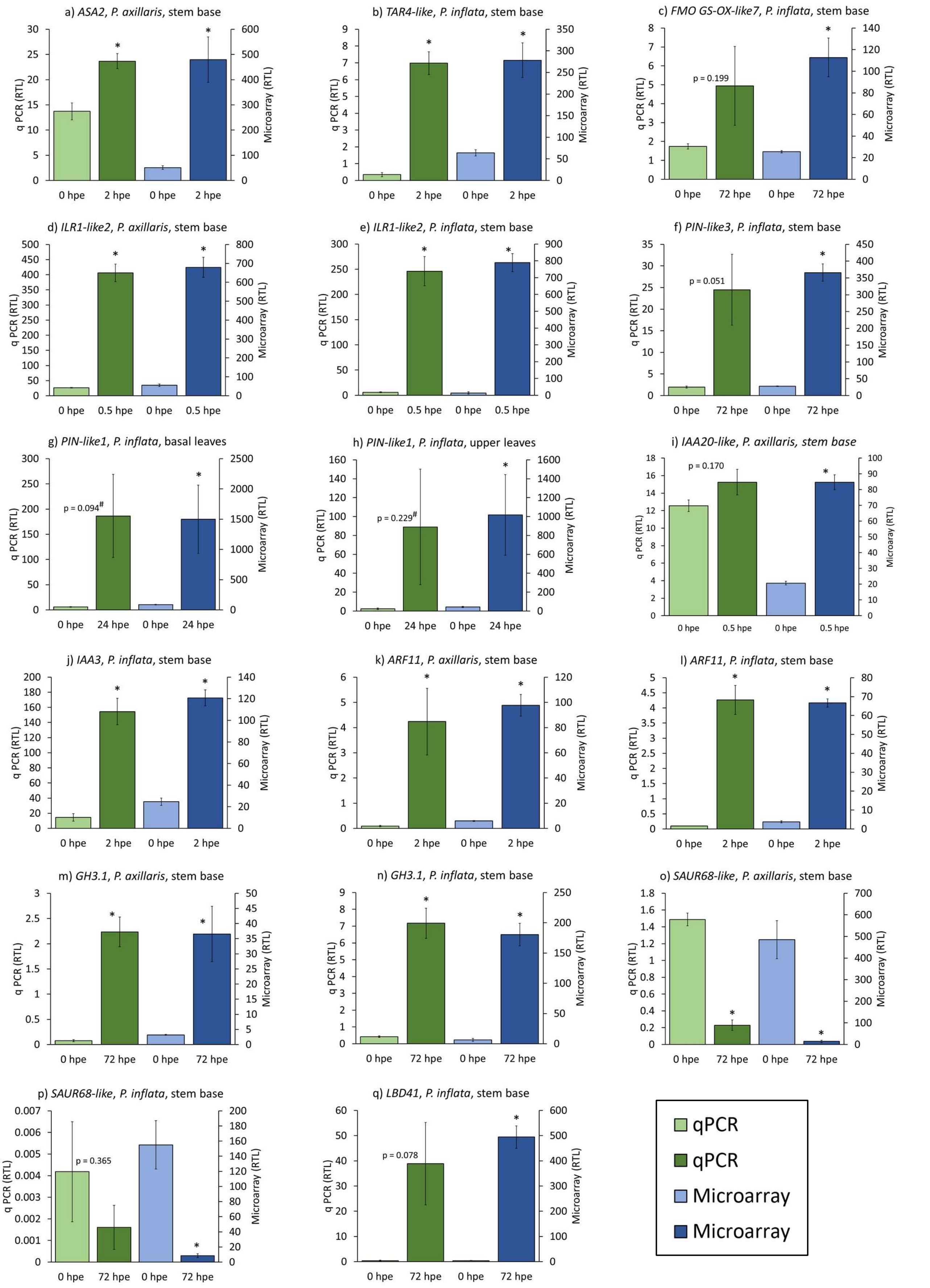

Supplementary Figure S2. Comparison of microarray and RT-qPCR results of transcriptome analysis of 17 selected auxin-related genes at selected time points. In case of RT-qPCR, relative transcript levels (RTL) were multiplied by 1000. Bars and whiskers show mean values ± SE. Asterisks indicate significant differences between the time points (t-test, n = 3, $p < 0.05$, each sample consisting of material from 10 or 16 cuttings for 24 and 72 hours post excision (hpe) or 0, 0.5 and 2 hpe, respectively). # in g) and h): for qPCR values, RTL at 24 hpe is significantly higher than RTL at 0 hpe (137.6 versus 7.1, $p < 0.05$, n =6), when the data of upper and lower leaves is calculated together.
