# Supplementary material for: Transcriptomic and hormonal dynamics in relation to adventitious rooting of two parental Petunia species highlight a coordinated activation of the jasmonate and auxin pathways and an important role of upper-shoot-derived auxin influx
Source: Front Plant Sci. 2026 Feb 6;16:1707238. doi: 10.3389/fpls.2025.1707238 (PMC12920520; doi:10.3389/fpls.2025.1707238)
Supplement: Supplementary file 3 [file DataSheet3.pdf]

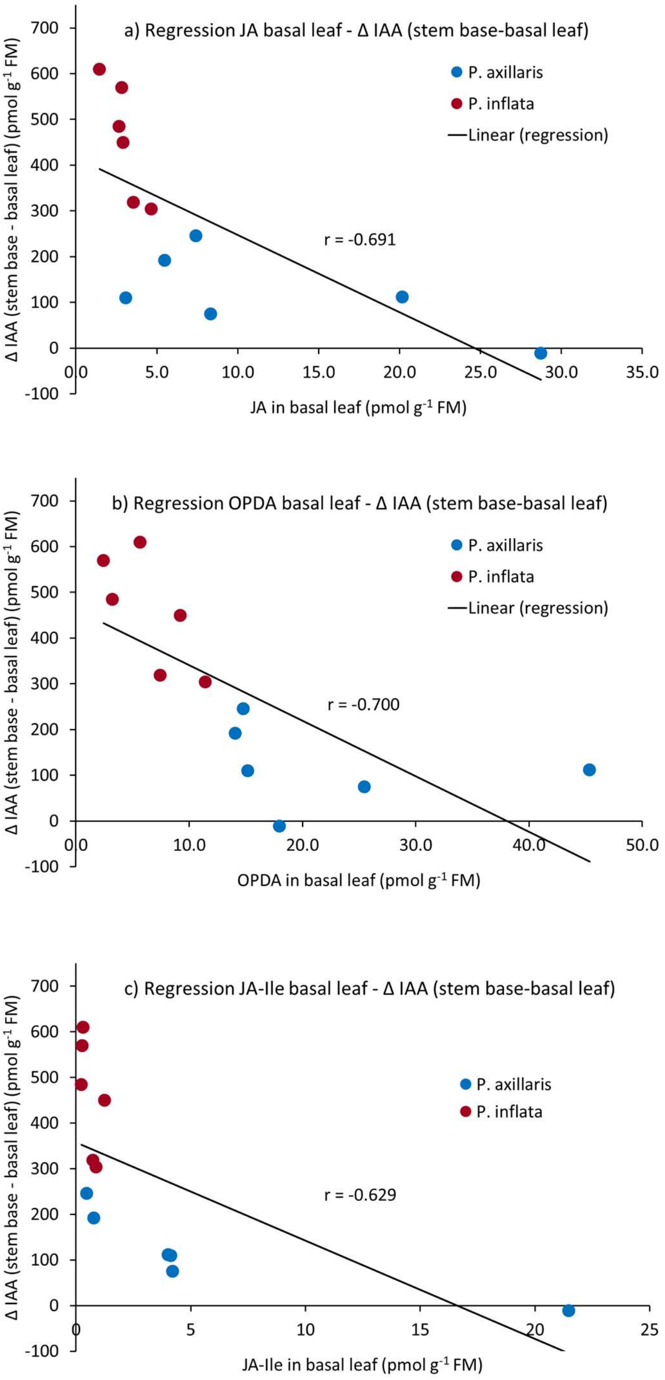

Supplementary Figure S3. Linear regressions calculated between JA(a), OPDA (b) and JA-Ile (c) in the basal leaf as independent variables and the IAA concentration gradient between the respective leaf and the stem base as dependent variable. Combined data of *P. axillaris* and *P. inflata* from 0 hpe and 24 hpe (n = 12). Each sample consisting of material from 10 or 16 cuttings for 24 hpe or 0 hpe, respectively.
